# Discrimination of Bipolar Disorder and Schizophrenia Patients Based on LC-HRMS Lipidomics

**DOI:** 10.3390/metabo16010069

**Published:** 2026-01-12

**Authors:** Milan R. Janković, Nataša Avramović, Zoran Miladinović, Milka B. Jadranin, Marija Takić, Gordana Krstić, Aleksandra Gavrilović, Čedo Miljević, Maja Pantović, Zorana Andrić, Savvas Radević, Danica Savić, Stefan Lekić, Vele Tešević, Boris Mandić

**Affiliations:** 1 University of Belgrade—Faculty of Chemistry, Studentski Trg 12–16, 11000 Belgrade, Serbia; mr.m.jankovic@gmail.com (M.R.J.); gkrstic@chem.bg.ac.rs (G.K.); vtesevic@chem.bg.ac.rs (V.T.); 2 Institute of Medical Chemistry, University of Belgrade—Faculty of Medicine, Višegradska 26, 11000 Belgrade, Serbia; 3 Institute of General and Physical Chemistry, Studentski Trg 12–16, 11158 Belgrade, Serbia; zmiladinovic@iofh.bg.ac.rs; 4 Department of Chemistry, University of Belgrade—Institute of Chemistry, Technology and Metallurgy, Njegoševa 12, 11000 Belgrade, Serbia; milka.jadranin@ihtm.bg.ac.rs (M.B.J.); danica.savic@ihtm.bg.ac.rs (D.S.); stefan.lekic@ihtm.bg.ac.rs (S.L.); 5 Group for Nutrition and Metabolism, Center of Research Excellence for Nutrition and Metabolism, University of Belgrade—Institute for Medical Research, National Institute of Republic of Serbia, Tadeuša Košćuška 1, 11000 Belgrade, Serbia; marija.takic@imi.bg.ac.rs; 6 Special Hospital for Psychiatric Diseases “Kovin”, Cara Lazara 253, 26220 Kovin, Serbia; gavrilovicaleksandra74@gmail.com; 7 Institute of Mental Health, University of Belgrade—Faculty of Medicine, Milana Kašanina 3, 11000 Belgrade, Serbia; cedo.miljevic@imh.org.rs; 8 Clinic of Psychiatry, University Clinical Center of Serbia, Pasterova 2, 11000 Belgrade, Serbia; pantovicm83@gmail.com; 9 Blood Transfusion Institute of Serbia, Svetog Save 39, 11000 Belgrade, Serbia; z.andric@nbti.org.rs (Z.A.); s.radevic@nbti.org.rs (S.R.)

**Keywords:** lipidomics, biomarkers, mass spectrometry, schizophrenia, bipolar disorder, serum

## Abstract

**Background/Objectives:** Schizophrenia (SCH) and bipolar disorder (BD) share overlapping symptoms and genetic factors, making differential diagnosis challenging and often leading to misdiagnosis. This study aimed to identify potential lipid biomarkers of serum capable of distinguishing BD from SCH. **Methods:** Lipid profiles of serum from 30 SCH and 31 BD patients were analyzed in triplicates using liquid chromatography–high-resolution mass spectrometry (LC-HRMS). Chemometric analysis was applied, including class and gender identifiers. Orthogonal partial least squares (OPLS) models with 1000 cross-validations were used to validate feature subsets. **Results:** The chemometric analysis included the most relevant metabolites in the comparison between all samples of SCH and BD patients, identifying five key biomarkers (LPC 16:0, SM 33:1, SM 32:1, compound C30H58O3, and PC 30:0) with VIP scores > 1 for distinguishing BD from SCH. Gender-specific models revealed five biomarkers in males (SM 32:1, SM 33:1, PC 32:1, PC 30:0, and FA 16:1) and two in females (LPC 16:0 and C30H58O3). These biomarkers primarily belonged to glycerophospholipids (GPs) and sphingophospholipids (SPs). **Conclusions:** Comparative lipid profiling between SCH and BD, including gender-specific subgroups, enabled identification of potential diagnosis-specific biomarkers. Elevated levels of GPs and SPs in SCH patients suggest lipid metabolism differences that may support improved diagnostic accuracy and personalized treatment strategies.

## 1. Introduction

Schizophrenia (SCH) and bipolar disorder (BD) are complex psychiatric diseases characterized by profound cognitive, behavioral, and emotional impairments [1,2,3]. They affect approximately 1% of the world’s population and their economic burden in the United States is estimated to exceed 300 billion dollars annually [4,5,6,7]. Based on the fact that the estimated burden of SCH in the United States doubled between 2013 and 2019 [8], effective strategies and treatment options are essential to improve the management of this disease. Schizophrenia and bipolar disorder arise from intricate interactions between genetic and environmental factors, and their underlying biological mechanisms are still not fully understood. The criteria for their psychiatric diagnoses are entirely based on observable symptoms, relying on the subjective assessments of psychiatrists following a structured clinical interview and patient or caregiver reports. This process is not only time-consuming but also limited by the clinician’s judgment. The shared symptoms among SCH and BD (with psychotic symptoms), such as delusions and hallucinations, further complicate accurate diagnosis. In addition to gaps in understanding and diagnosing these disorders, there is an urgent need for personalized, targeted approaches to treatment, including individualized medical therapies and reliable methods for monitoring treatment effectiveness [9,10].

‘Omic’ research, which analyzes a wide range of biomolecules within biological samples, has significant potential for uncovering new diagnostic markers that could pave the way for better identification and treatment of SCH and BD. These biomarker sets could be developed using advanced techniques such as nuclear magnetic resonance (NMR), liquid chromatography-mass spectrometry (LC-MS) and gas chromatography-mass spectrometry (GC-MS) [11,12,13,14,15,16,17]. Metabolomics and lipidomics analyses, combined with chemometric assessments of serum and urine samples from both patients and healthy individuals, could provide valuable insights [12,13,14,15,16,17,18,19,20]. In recent years, chemometric methods have been increasingly developed to help extract useful information from lipidomic data, playing a significant role in the identification and validation of potential biomarkers and contributing to improved disease diagnosis and monitoring [18,19,20,21,22,23,24,25,26].

Lipids comprise a heterogeneous group of molecular subclasses that exert essential regulatory functions in neuronal development and activity, modulate membrane-associated neuronal plasticity [27,28,29], and influence brain energy metabolism [30,31,32].

Previous LC-MS studies on schizophrenia [18,19,21,22,23] and bipolar disorder [20,24,25,26] indicate that potential lipid biomarkers may belong to various serum and plasma lipid categories, including glycerophospholipids (GP), sphingolipids (SP), glycerolipids (GL), sterol lipids (ST), and fatty acyls (FA). Several recent lipidomics studies suggest that antipsychotic treatments in SCH patients could lead to the alteration of serum lipid profiles, including the downregulation of shorter-chain triacylglycerols [21,33,34]. Diverse lipidomics studies have highlighted the strong influence of lipid metabolism in SCH and BD [18,19,20,21,22,23,24,25,26]. For instance, abnormalities in phospholipid metabolism have been strongly linked to SCH [18,19,21,22,23], while lower levels of omega-3 polyunsaturated fatty acids have been observed in individuals with both SCH and BD, suggesting possible therapeutic applications [35,36]. However, no biomarkers have yet been approved for diagnosing psychiatric disorders.

Our research group recently studied lipid profiles of SCH (30) and BD (31) patients compared to their controls (SCH-C 31 and BD-C 31), applying liquid chromatography coupled with high-resolution mass spectrometry (LC-HRMS) [19,20], and established the alteration of the majority of lipid classes: GP, SP, GL, fatty acids (FA), and cholesterol esters (CE), indicating a key role of lipid pathways in the pathogenesis of SCH and BD. These findings about the alterations of lipid profiles in SCH and BD could enable the discovery of potential biomarkers, enhance SCH and BD diagnosis, and support the development of more effective treatments [18,19,20,21,22,23,24,25,26].

The aim of this work is to compare the lipid profiles of sera from SCH and BD patients and explore the potential for their differentiation by combining obtained LC-HRMS-based lipid data with chemometric analysis.

## 2. Materials and Methods

### 2.1. Sampling

This study followed ethical guidelines from Kovin Psychiatric Hospital and the University of Belgrade Faculty of Chemistry. Blood samples were collected from 30 SCH patients and 31 BD patients at Kovin Hospital. All participants or their guardians gave written consent. No statistically significant differences were found between the two groups of patients in terms of age, gender, or BMI. SCH patients (100%) were treated with anxiolytics, while 6.7% used first-generation antipsychotics, 50% second-generation, and 43.3% both. BD patients were treated with first-generation antipsychotics (6.45%), second-generation antipsychotics (77.42%), and anxiolytics (16.13%). Blood serum samples were collected after at least 8 h of fasting and each sample was prepared in triplicate for analysis.

### 2.2. Sample Preparation

Blood samples were kept on ice for one hour, then centrifuged. Serum was collected and stored at −80 °C for up to two weeks. Lipids were extracted in triplicate following O’Brien et al. (2019) [37]. LC–HRMS analysis and data processing of lipid extracts of bipolar disorder (BD) and schizophrenia (SCH) patients, together with extraction blanks and pool samples, were performed with slight modifications as described in Jadranin et al. (2023) [20].

### 2.3. Software

Data processing and chemometric analysis were performed using MATLAB 9.7 (MathWorks, Natick, MA, USA) [38] with suitable toolboxes and in-house routines. The subroutine for the Borda count method was adapted from Python’s mlpy module version 3.0 [39], while LC-HRMS chromatograms and spectra were processed using the R package XCMS 3.16.1 [40] and then imported into MATLAB workspace. The Robust PCA (ROBPCA) method [41,42] from the LIBRA package [43] was used alongside classical PCA for efficient outlier detection. Preprocessing and chemometric analysis were conducted with PLS Toolbox 8.9.1 [44].

### 2.4. Reading in Data

After the cleaning of background noise, and isotopic and unrelated ions, the initial dataset included 183 chromatograms: 93 triplicates from 31 individuals with BD and 90 triplicates from 30 individuals with SCH, and 183 variables (featuring or one or more ions per metabolite with characteristic *m*/*z* and retention time values) belonging to the 129 metabolites for combined negative–positive ions. Additional categorical variables, such as class, gender, triplicate identifiers, and grouping, were included in final dataset structure [19,45,46]. Replicated samples were used to identify different acquired LC-MS chromatograms originating from the same individuals, which also facilitated feature validation.

### 2.5. Data Pretreatment (Preprocessing)

Before further processing, data pretreatment steps were necessary, including handling missing data through removal or imputation, data transformation, normalization, and the final centering and scaling of all variables. Since centering and scaling depend on sample size, these steps were applied before model assembly and cross-validation. Excluding observations with missing values results in further dataset reduction; hence, replacement or imputation represent a more reliable approach in situations where a relatively low number of observations are present in the dataset [19]. Linear interpolation of neighboring non-missing values was most suitable for this dataset [47], though a moving median (over a 3-point window) produced similar outcomes. As result, all identified missing values were handled using linear interpolation.

For LC-MS metabolomics data, performing logarithmic transformation (e.g., replacing each value x with log10(x) or log2(x)) [48,49] reduced extreme values, producing homoscedastic and near-normal residuals. Figure S1a (Supplementary Materials) shows boxplots of log10-transformed data grouped by classes (“SCH” for schizophrenia and “BD” for bipolar disorder), while Figure S1b (Supplementary Materials) presents the same data, mean-centered after logarithmic transformation.

Scaling is often paired with centering, with “autoscaling” being the most common method. Autoscaling involves mean centering each metabolite’s data (subtracting the sample mean) and dividing by the standard deviation [48], ensuring a unit standard deviation for unbiased comparisons. The log10-transformed results in Figure S1a (Supplementary Materials) show significantly reduced skewness compared to untransformed data. Additionally, Figure S1b (Supplementary Materials) highlights the benefits of mean centering and scaling to the standard deviation for future modeling.

Hence, this study applies consistent log10 transformation followed by autoscaling (mean centering and scaling to standard deviation) to all variables in the dataset.

### 2.6. Data Normalization

Principal Component Analysis (PCA) of the samples processed across four consecutive batches (Figure S2, Supplementary Materials) revealed no significant drift. Additionally, the comparison of intra-day and inter-day coefficients of variation (CV) for filtered *m*/*z* (rt) values in quality control (QC) samples showed CVs below 30%. Therefore, fluctuations in instrument sensitivity during measurements were deemed negligible, and no further normalization of the samples was performed.

### 2.7. PCA and Outlier Detection/Identification

Classical PCA was applied to the initial datasets (overall and gender-specific subsets), but it revealed no significant variation between groups or classes. However, classical PCA, combined with robust PCA (ROBPCA), was used for outlier identification. ROBPCA outlier maps, based on score and orthogonal distances, identified “bad leverage points” as relevant outliers. Screeplots (LIBRA package) determined the number of PCA components for each dataset for the whole dataset, seven samples (33, 48, 110, 111, 130, 175, and 176) were flagged as outliers (Figure S3a, Supplementary Materials). Outlier maps for gender-specific subsets (Supplementary Materials Figure S3b,c) revealed outliers: three samples (33, 65, and 66) in the male subset, and three samples (3, 91, and 92) in the female subset. All identified outliers based on this method (robust PCA) for gender-specific subsets match with corresponding outliers identified for the complete dataset. Further analysis with classical PCA identified one additional outlier in each gender-specific subset. This way, a total of seven distinctive outliers were identified in the main dataset, with four outliers in each of the gender-specific subsets (male and female participants). Figure S4a,b show the skewness and kurtosis results before and after removing the seven outliers from the main dataset.

The results in Figure S4 (Supplementary Materials) show a significant reduction in skewness and kurtosis after removing the identified outliers, particularly in the *m*/*z* range of 675–850. Consequently, all identified outliers were excluded from the corresponding datasets and subsets prior to further LC-MS data analysis.

### 2.8. Cross-Validation (CV) and Feature Validation Dataset Partition

In this work, a 5-fold contiguous block CV method was used, keeping triplicates from the same patients in the same block. CV blocks were formed by splitting the dataset and randomly shuffling samples within each block while maintaining triplicate structures [45]. For thorough feature validation, an additional observation subsampling approach was applied. For each CV fold group, one triplicate per sample was randomly selected. The subsampling process involved three steps: (1) partitioning the dataset into folds, grouping samples with their triplicates (repeated *iter1* times); (2) for each fold group, selected samples are gathered samples from all other remaining folding groups to create *n* groups of observations for further analysis; and (3) randomly sub-selecting one triplicate per sample in each fold (repeated *iter2* times). The final matrix of logical indices of selected observation had dimensions (*iter1* × *iter2* × *fold*, *N*), where *N* is the total number of observations of each subset, and fold represents the number of used fold groups. For the current investigation following parameter settings were consistently employed: *iter1* = 100, *iter2* = 500, and fold was set to 5 in all cases. Full details are available in [19] and the Supplementary Materials of the current work.

### 2.9. Classification Performance Metrics for Model Evaluation

To compare classification performance across models, various performance metrics can be utilized. Selecting and interpreting the right metrics is crucial for accurate model evaluation, as relying on a single metric might not suffice. Instead, combining metrics provides better insights for model fine-tuning during development.

In this study, the OPLS-DA model was evaluated using a confusion matrix derived from cross-validated predicted class labels. Metrics such as accuracy, sensitivity, and specificity were calculated from the matrix:

Accuracy: The ratio of correctly classified samples to total samples, especially effective for near equally balanced datasets.
Sensitivity: Depends on correctly identifying the SCH group as positive samples.
Specificity: Relies on accurately classifying the BD group as negative samples.

In all cases, SCH was treated as the positive class and BD as the negative class. These metrics were specifically expressed for this study based on this classification framework:

$$Accuracy=\frac{T_{SCH}+T_{BD}}{T_{SCH}+T_{BD}+F_{SCH}+F_{BD}}$$

$$Sensitivity=\frac{T_{SCH}}{T_{SCH}+F_{BD}},\;Specificity=\frac{T_{BD}}{F_{SCH}+T_{BD}}$$

Correctly classified SCH samples are marked as *T_SCH_*, while those misclassified as BD are *F_BD_*. Similarly, BD samples are counted as *T_BD_* if correctly classified, and *F_SCH_* if misclassified.

Accuracy, though widely used, is sensitive to imbalanced datasets where one class outnumbers the other [50]. In this study, the SCH to BD ratio ranges from 1 (entire dataset) to 1.22 (male) and 0.86 (female), ensuring the least-populated class represents no less than 45% of the data. However, using metrics insensitive to class imbalance is a safer choice when multiple methods are evaluated together.

Apart from sensitivity and specificity, which are robust to imbalance, another complementary metric is class error (or Balance Error Rate/Half Total Error Rate) [50,51]. This uses 1-*sensitivity* and 1-*specificity* for evaluation.

$$classerror=1-\frac{\left(sensitivity+specificity\right)}{2}=\frac{1}{2}\left(\frac{F_{SCH}}{F_{SCH}+T_{BD}}+\frac{F_{BD}}{T_{SCH}+F_{BD}}\right)$$

The AUROC (Area Under the ROC Curve) is a metric used to numerically compare classifiers by reducing the ROC curve to a single scalar value that represents classification performance [52]. In this study, AUROC was calculated using cross-validated predictions and reflects the probability that the OPLS-DA classifier ranks a randomly chosen SCH instance higher than a BD instance [48]. A perfect classifier yields an AUROC of 1.0, while an AUROC of 0.5 indicates random classification. No classifier realistically falls below 0.5 [50].

While PLS-DA is a classification model, it is fundamentally based on the PLS regression algorithm [53,54,55]. Additionally, RMSECV (Root Mean Square Error by Cross-Validation) was introduced for continuity with prior publications [19,45] and to compare its diagnostic utility alongside classification metrics in this study.

## 3. Results

### 3.1. OPLS-DA Models

**Figure 1:**
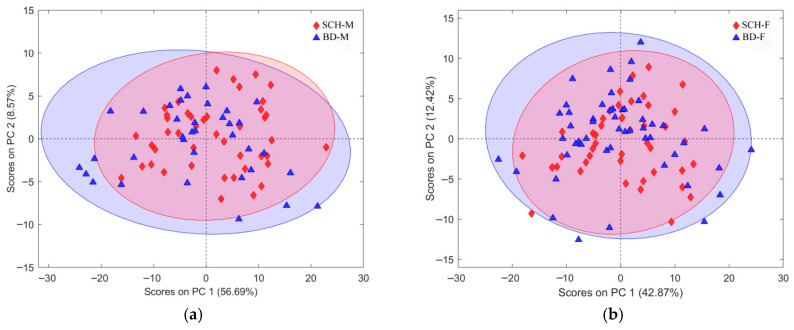
PCA model for dataset partitions with: (**a**) male subjects and all belonging features, using four PCA components; (**b**) female subjects and all belonging features, using four PCA components. Scores plots present first two PC components with assigned explained variability for each of them. Each group of patients was assigned a different color, BD as blue full triangles and SCH as red full diamonds.

In assembled PCA models, no distinctive patterns were observed among scores for the class of interest, consistent with their unsupervised nature and limited utility for biomarker identification [48] (results for gender-specific datasets are depicted in Figure 1). PCA performs best when among-group variability exceeds within-group variation. In contrast, supervised methods like PLS-DA and OPLS-DA better highlight class distinctions by linking predictors to outcomes [53,56,57], emphasizing within-class similarities, and at the same time, between-class differences. To avoid overfitting, models were validated via cross-validation and external testing [56,58]. OPLS-DA parameter optimization often involves mutually combining different CV methods and model performance metrics. To convert the obtained output of OPLS-DA self-predicted continuous values of responses into reliable binary labels, thresholds for each class were determined using a Bayesian method [51,59,60].

In addition to the developed cross-validation (CV) method (detailed explanation in the Experimental section) for datasets containing triplicates, venetian blinds and random subset-selected CV methods [61] were also applied. Determining the optimal model complexity, such as the number of latent variable (LV) components, is a critical step for PLS-DA modeling. For this study, a random sampling and further sub-selection method was used to identify the most reliable number of LV components for model assessment. The procedure can be summarized as follows: (1) Randomly partition the main dataset into 5-fold groups of observations, maintaining triplicate structures. Sub-partitioning by male and female groups was also performed during optimization. (2) Independently sub-select one triplicate per sample within each fold, repeating this 1000 times.

**Figure 2:**
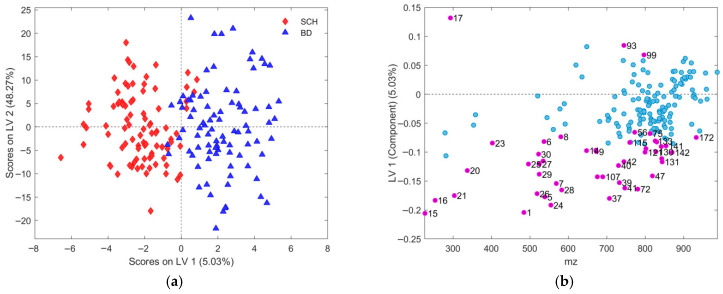
(**a**) Score plot of the OPLS-DA model composed of the dataset from all observations with excluded outliers, where discrimination classes belong to the BD (blue full triangles) and SCH (red full diamonds) groups of samples. (**b**) Loading plot of the predictive component LV1, where VIP scores > 1 determined from the model are presented in pink with assigned feature position numbers from the corresponding dataset positions. The accuracy was 0.8523, as well as the sensitivity and specificity, too, for this model. AUROC was determined to 0.9401.

For each subset, OPLS-DA models with up to 10 LV components were generated. The optimal number of LV components was determined based on the minimum RMSECV (Root Mean Square of Error by Cross-Validation), minimum class error, and maximum AUROC values. Figure S5 in Supplementary Material presents histograms for each fold, showing sub-selected samples with one triplicate and “the venetian blinds” selection method applied during CV. As shown in Supplementary Materials Figure S5, three LV components were most frequently selected, providing optimal model performance across the selected metrics. Additionally, permutation tests confirmed this result, indicating that models with more than three LV components are prone to overfitting (see Supplementary Materials Figure S6 for the OPLS-DA model in Figure 2). Consequently, all OPLS-DA models in this study were constructed using three LV components.

**Figure 3:**
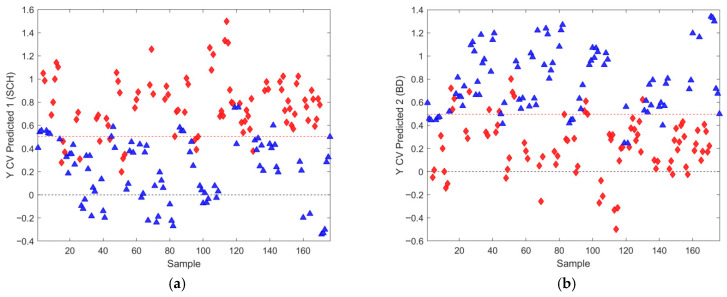
For the best selected OPLS-DA model presented in Figure 2, we present results for: (**a**) Cross-validated prediction results for class SCH (red filled diamonds), where the discrimination threshold was determined at 0.5036 (red dashed line) (**b**) Cross-validated prediction results for class BD (blue filled triangles), where the discrimination threshold was determined at 0.4964 (red dashed line).

Figure 2 presents the scores and loading plots of the predictive LV1 component for the best OPLS-DA model identified among 1000 models generated during CV, all using three latent variables (LV) and a dataset with all observation with outliers previously removed. The results for accuracy, sensitivity, and specificity for this model are 0.8523, whereas AUROC was 0.9401 at the same time. Variance captured along the predictive LV component was 5.03%, which, together with different performance metric distributions (details presented in Section 3.4), suggests the relatively poor performance of these models. Additionally, CV prediction results for both patient classes are presented in Figure 3.

### 3.2. Feature Selection and Validation

Selection of features (two-dimensional (*m*/*z* and retention time) LC/MS signals or ions) and their validation are essential for building efficient predictive models by identifying relevant variables and reducing dimensionality [62]. Variable Importance in Projection (VIP) is a commonly used method, with the “greater than one” rule (VIP > 1) serving as a typical selection threshold [63]. However, values between 0.83 and 1.21 may be more suitable in specific cases [64,65,66]. Stability can be improved through the bootstrap with replacement resampling technique, which produces multiple VIP estimates per variable [65]. Larger values for resampling enhance the handling of unsmooth and nonlinear data, but at the cost of increased computational time.

In our previous study [19], a similar approach with a slightly modified sub-sampling method based on k-fold partition of the dataset was introduced. For the present study, the procedure for feature selection and subsequent validation was slightly adopted and upgraded, detailed in the Supplementary Materials (*Features selection methods and algorithm*) [19,37,67,68].

Feature validation was extended by applying feature selection before each validation step. Features are ranked by VIP scores, the least informative ones are removed, and the remaining set is evaluated using an OPLS-DA model. This cycle repeats until optimal performance is reached [66]. The approach resembles stepwise backward elimination [66,69], with modifications that leverage the ranked VIP score list.

To evaluate OPLS-DA model performance in relation to the number of included features, feature validation plots were constructed using classification metrics derived from cross-validation (CV) confusion matrices and RMSECV. Accuracy and AUROC were used to estimate classification performance [48,50], while RMSECV and class error were plotted as loss curves. These curves provided insight into how model performance evolved by measuring classification error over cumulative feature subsets from the ranked list.

Validation curves were generated across cumulative subsets of ranked features. These typically exhibited a sharp initial performance increase [70], followed by an extreme point, maximum for accuracy and AUROC, minimum for RMSECV and class error, and a subsequent decline, often attributed to the inclusion of noise variables. Irregular patterns in the early curve regions, such as sporadic local minima or maxima, suggested the presence of less informative features and were considered during further subsetting refinement.

**Figure 4:**
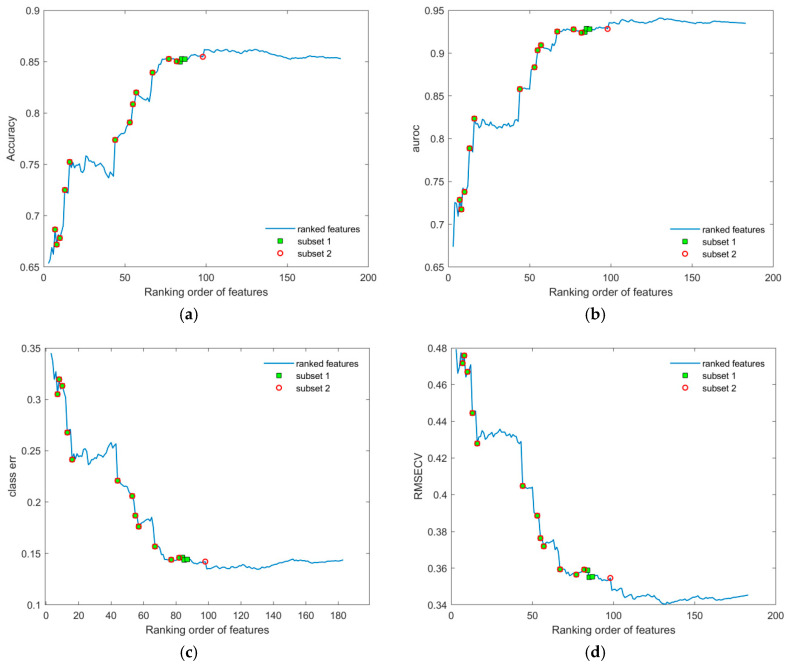
Several performance metrics are displayed in relation to the increasing sequence of sets of ranked features for the initial dataset (the number of features in the subsets that were created by adding features cumulatively, one by one, from the ranking list): (**a**) accuracy; (**b**) AUROC; (**c**) class error; and (**d**) RMSECV. Additionally, markers colored as green squares (subset 1) and red circles (subset 2) represent the smallest sets of features identified through the subsequent selection procedure (shown in Figure 6 and Table 1), and are included for comparison with the original feature list. The first two features were omitted, since the number of LV components was always 3 for each assembled OPLS-DA model during validation.

**Figure 5:**
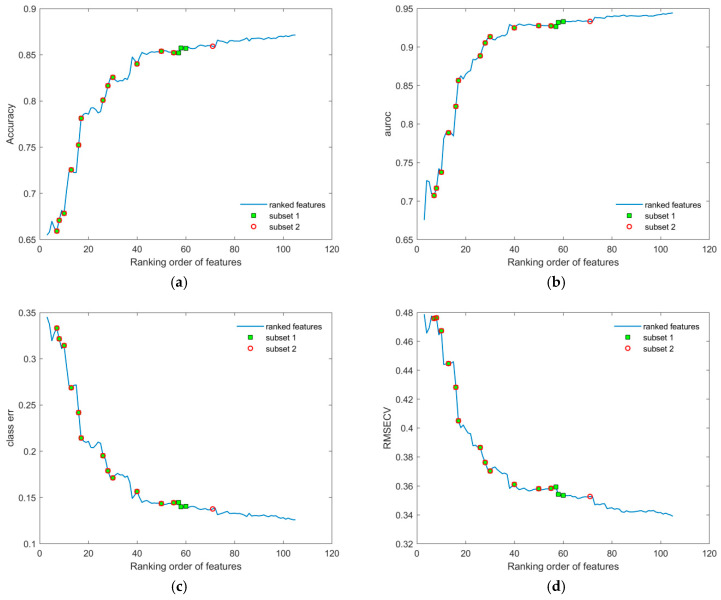
Performance metrics displayed in relation to the increasing sequence of sets of ranked features of the initial dataset: (**a**) accuracy; (**b**) AUROC; (**c**) class error; and (**d**) RMSECV. Additionally, markers colored as green squares (subset 1) and red circles (subset 2) represent the smallest sets of features identified through the subsequent selection procedure (shown in Figure 6 and Table 1), and are included for comparison with the original feature list. The first two features were omitted, since the number of LV components was always 3 for each assembled OPLS-DA model during validation.

Model performance was evaluated across the full dataset, including both male and female subjects, using complete sets of features ranked by VIP scores (see *Features selection and validation* in Supplementary Materials). As shown in Figure 4, performance metrics exhibited a distinct peak around a subset of ~130 features, while a plateau was observed between ranks 17 and 43. Variables within this plateau were repositioned to the start of the sequence and re-evaluated, revealing their limited contribution to all model performance metrics. These features were subsequently excluded. The revised validation curves, shown in Figure 5, demonstrate a smoother and more consistent increase in performance across all metrics, with reduced sporadic disruption compared to the original ranked list. This suggests that careful feature selection and exclusion of low-impact variables can significantly enhance model robustness, on which validation curve results depend.

**Figure 6:**
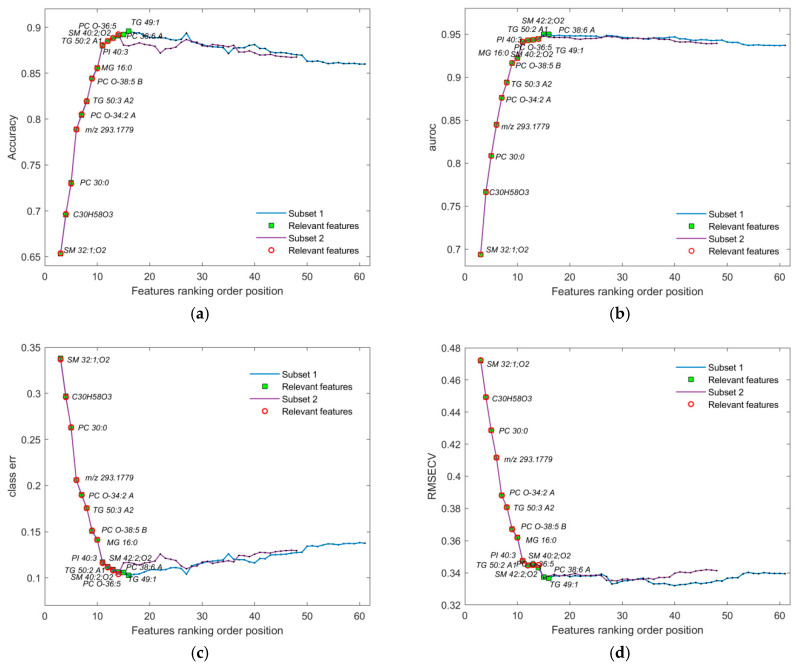
Performance metrics displaying the smallest subset of the identified subset of features for the initial dataset: (**a**) accuracy; (**b**) AUROC; (**c**) class error; and (**d**) RMSECV. Markers colored as green squares represent subset 1, consisting of the first 16 most relevant features out of the 61 total presented features on the plot. Markers colored as red circles represent subset 2, consisting of the first 14 most relevant features from the 48 total presented features. The first two features were omitted, since the number of LV components was always 3 for each assembled OPLS-DA model during validation. The figure results preview the data presented in Table 1.

**Table 1:** For the full dataset, a list of identified relevant features for subset 1, consisting of the 16 most relevant features from the 61 total features in this subset; and for subset 2, consisting of the 14 most relevant features from the 48 total features in this subset.

Subset 1		Subset 2	
ID Number	Feature Names	ID Number	Feature Names
24	LPC 16:0 A1	24	LPC 16:0 A1
107	SM 33:1;O2	107	SM 33:1;O2
106	SM 32:1;O2	106	SM 32:1;O2
1	C30H58O3	1	C30H58O3
37	PC 30:0	37	PC 30:0
17	*m*/*z* 293.1779	17	*m*/*z* 293.1779
93	PC O-34:2 A	93	PC O-34:2 A
144	TG 50:3 A2	144	TG 50:3 A2
101	PC O-38:5 B	101	PC O-38:5 B
35	MG 16:0	35	MG 16:0
103	PI 40:3	103	PI 40:3
140	TG 50:2 A1	140	TG 50:2 A1
118	SM 40:2;O2	118	SM 40:2;O2
123	SM 42:2;O2	98	PC O-36:5
82	PC 38:6 A		
135	TG 49:1		

To improve the resulting performance, we applied additional subsetting to the ranked feature list by removing noisy or redundant features. This approach, illustrated in Figure 6, significantly reduced computational load, particularly given the high cost of validation (e.g., 1000 cross-validation iterations per point). The final point in the sequence with the corresponding performance metric within each reduced subset was used as a benchmark for evaluating overall performance for the current list of ranked features.

Iterative pruning revealed the features that consistently degraded performance. Their removal led to measurable improvements in all performance metrics and stability. However, in the cases where performance declined after the removal of a particular feature, reverting to the previous optimal configuration step was necessary. Furthermore, reordering features around critical thresholds exposed additional low-impact features, enabling further refinement of the feature set.

The smallest subsets of features (1 and 2) identified through the subsequent selection procedure (Figure 4, Figure 5 and Figure 6), together with the corresponding feature coding lists, are presented in Table 1. Their more detailed identification can be found in Table S1 (Supplementary Materials).

### 3.3. Data Subset with Male Participants

The same approach of the selection procedure as in the previous Section 3.2 also enabled subgroup-specific reductions, such as for male and female subjects. Figure S7 (Supplementary Materials) shows various model performance metrics across increasing subsets of features for male participants. The features range from N (the number of LV components in the current OPLS-DA model) to all 183 features included in the data subsets. The validation plot in Figure S7 highlights a features sequence region between 11 and 60 features, with an extreme used performance value near the 130th feature. Features beyond this point, and within the identified region, are strong candidates for elimination using the previously described subsetting techniques. Notably, the first eight features show a sharp improvement in classification performance, based solely on their frequency positions in the general ranked list (see the aggregation methods for ranking presented in Section 3.2, Supplementary Materials).

**Figure 7:**
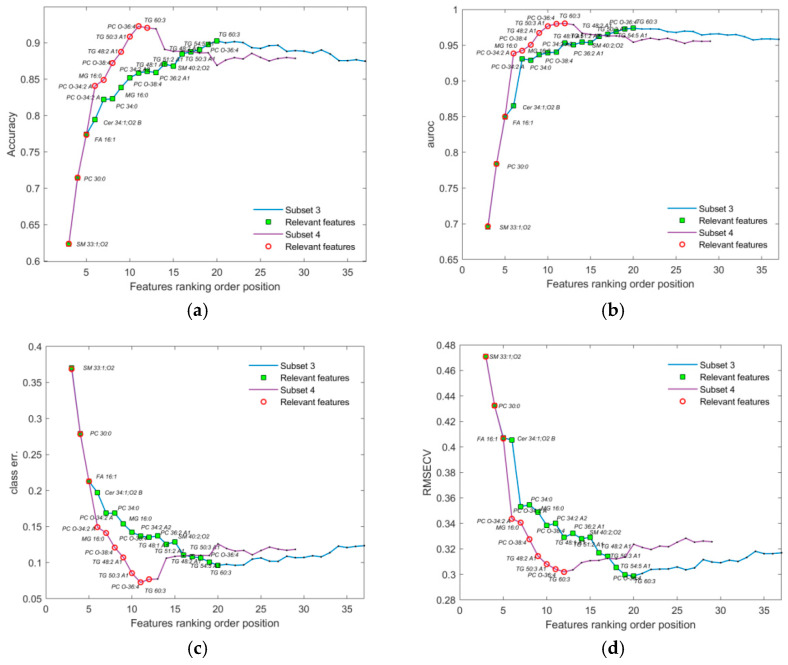
Performance metrics displaying the smallest subset of the identified subset of features for partition of the initial dataset containing male subjects only: (**a**) accuracy; (**b**) AUROC; (**c**) class error; and (**d**) RMSECV. Markers colored as green squares represent subset 3, consisting of the first 20 most relevant features out of the 37 total presented features on the plot. Markers colored as red circles represent subset 4, consisting of the first 12 most relevant features from the 29 total presented features. The first two features were omitted, since the number of LV components was always 3 for each assembled OPLS-DA model during validation. Both subsets with corresponding feature coding lists are given in Table 2.

Through feature subsetting, the smallest subset (subset 4) providing the best performance metrics was identified. The validation plot in Figure 7 compares subset 4 with an intermediate subset (subset 3), which contains a few additional features but shows worse average performance for all OPLS-DA model metrics. Subset 4, consisting of 12 relevant features, is presented alongside subset 3 in Table 2. Their more detailed identification can be found in Table S1 (Supplementary Materials).

### 3.4. Data Subset with Female Participants

Figure S8 (Supplementary Materials) presents the model performance metrics for the increasing feature subsets among female subjects, filtered from the initial dataset. The features are arranged in the same way as in the previous datasets and observations.

Inspection of the validation plot reveals a declining trend in classification performance metrics for the first ten feature sequences. Erratic performance is observed in the regions between positions 15–23 and 37–38 of the global ranked features. An extreme metric value is identified for the sequence of the first 46 features. As a first step, features 47 to 183 were excluded from the next subsetting list. Additionally, the first ten features were rearranged and repositioned to identify better candidates for elimination before proceeding further.

**Figure 8:**
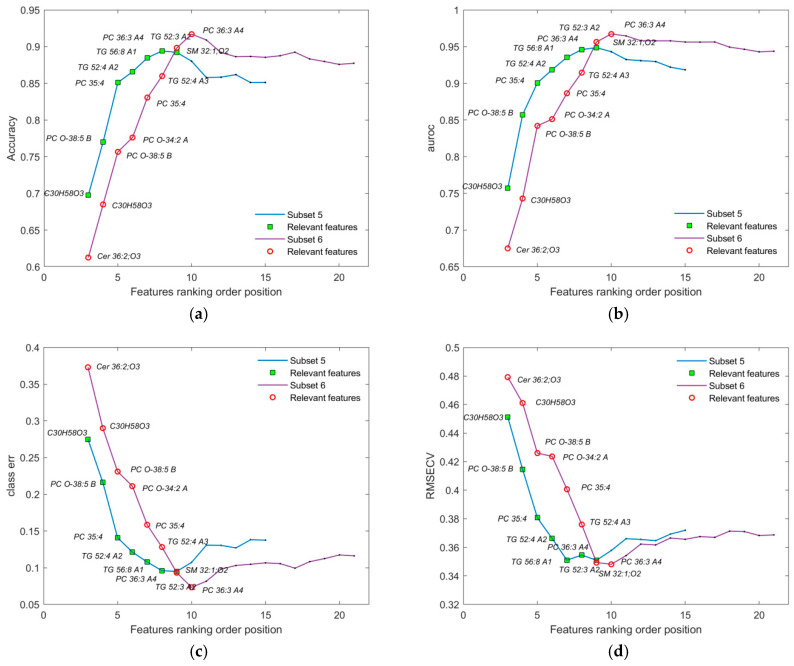
Performance metrics displaying the smallest subset of identified subset of features for partition of initial dataset containing only female subjects: (**a**) accuracy; (**b**) AUROC; (**c**) class error; and (**d**) RMSECV. Markers colored as green squares represent subset 5, consisting of the first 9 most relevant features out of the 15 total presented features on the plot. Markers colored as red circles represent subset 6, consisting of the first ten most relevant features from the 21 total presented features. The first two features were omitted, since the number of LV components was always 3 for each of the assembled OPLS-DA models during validation. Both subsets with corresponding feature coding lists are given in Table 3.

After feature subsetting, the smallest subset with the best performance metrics was identified. Figure 8 illustrates this process, showing two selection pathways: subset 5 and subset 6. Subset 5 contains 9 relevant features out of 15, but performs worse across all classification metrics compared to subset 6. Subset 6, with 10 relevant features out of 21, demonstrates superior performance for the selected features. A detailed list of identified features for both subsets is provided in Table 3, and their more detailed identification can be found in Table S1 (Supplementary Materials).

### 3.5. Comparison of Model Performance Metric Distribution

Model performance metrics (accuracy, AUROC, sensitivity, and specificity) were compared across 1000 independently generated CV OPLS-DA models using either all features or selected subsets (Table 1, Table 2 and Table 3; Supplementary Materials Figures S9–S11). Median values (dashed lines) for corresponding distribution plots revealed improved overall performance with feature selection.

During validation, each point in the plots (Figure 4, Figure 5, Figure 6, Figure 7 and Figure 8) represents the mean of the corresponding metric distributions. Mean metric values were aggregated from 183 feature sequences (or fewer during subsetting) with 1000 outcomes. Wilcoxon rank-sum tests (1% significance level) indicated significant increases in accuracy, AUROC, sensitivity, and specificity (left-tailed), and significant decreases in RMSECV and class error (right-tailed) [71].

Despite overall improvements, distribution plots showed slight negative skewness for models with selected features, suggesting marginally reduced performance in some cases. Skewness for the datasets including all observations ranged from −0.1 to −0.7 for the full and reduced feature sets, with stronger skewness in gender-partitioned data (e.g., AUROC for males: −2.6; specificity for females: −1.75; Table 4).

A plausible explanation for such behavior, at least for the former distribution corresponding to AUROC for male subjects presented in Supplementary Materials Figure S10b, could be that the distribution is truncated on the right side with an upper limit equal to 1; this is also relevant for all other distributions with given metrics where truncation was bonded within the [0, 1] interval. In contrast, all other distributions with moderate observed skewness could not be fitted with a truncated normal distribution, since they result in the same average values for the corresponding metric distribution as the untruncated normal distribution.

This skewness may also result from outliers emerging under reduced feature sets, undetected in full-feature PCA, and from diminished predictive power in certain samples.

### 3.6. Performance of OPLS-DA Models with Selected Feature Subsets

From 1000 CV OPLS-DA models built for each dataset and feature subsets (Table 1, Table 2 and Table 3), the best model was determined based on the most frequent metric range on any given distribution (accuracy, sensitivity, and specificity). The optimal model, also exhibiting the best AUROC, was identified using multiple intersects [72]. The specific metric performance for these models is summarized in Table 5.

**Figure 9:**
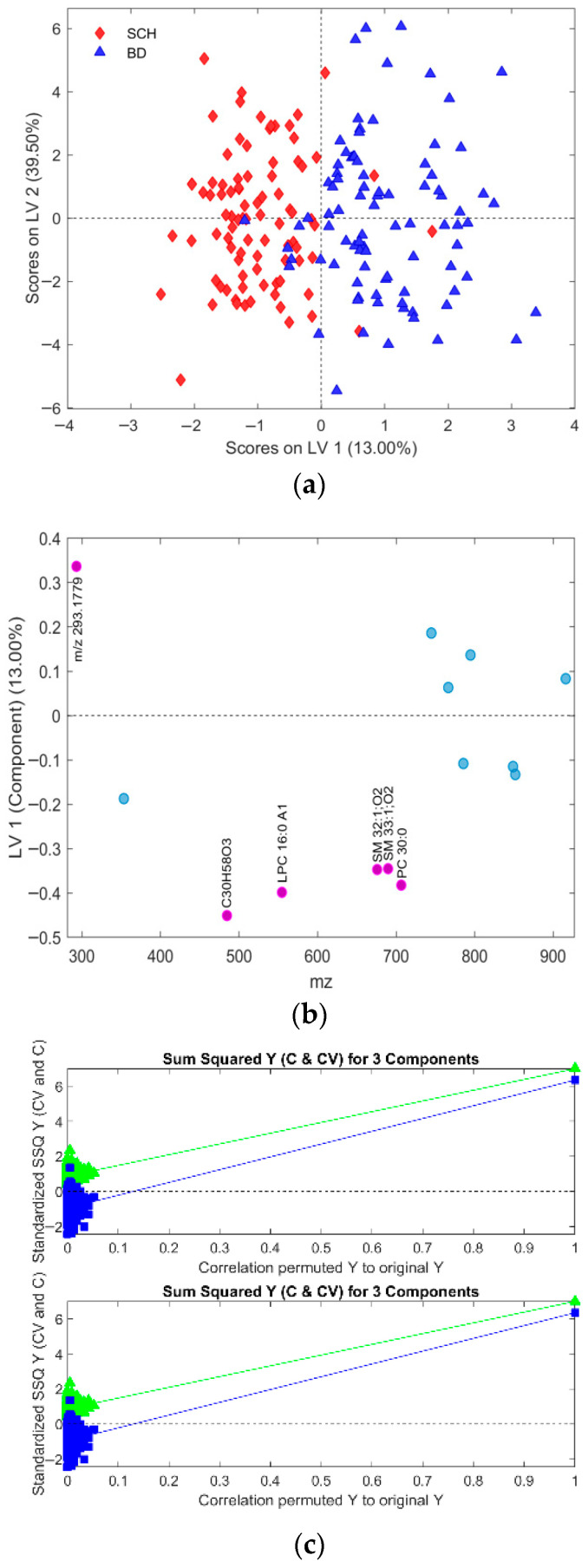
(**a**) Scores plot of the first two LV components of the OPLS-DA model using mean centering and unit variance scaling. The variance captured by the first two LV components, predictive LV1 and orthogonal LV2, is given in the figure axis labels. (**b**) Loading LV1 component of the PLSDA model for the set of features assigned as subset 2 (given in Table 1), with the relevant set of features including all initial samples with identified outliers excluded, where features with VIP scores greater than 1 are indicated as pink circles assigned with corresponding names. (**c**) Permutation test for the OPLS-DA model composed of three LV components and the set of selected features assigned as subset 2, performed with 500 iterations. Fractional y-block information captured by calibration (green) and cross-validation (blue) versus y-correlation is shown.

**Figure 10:**
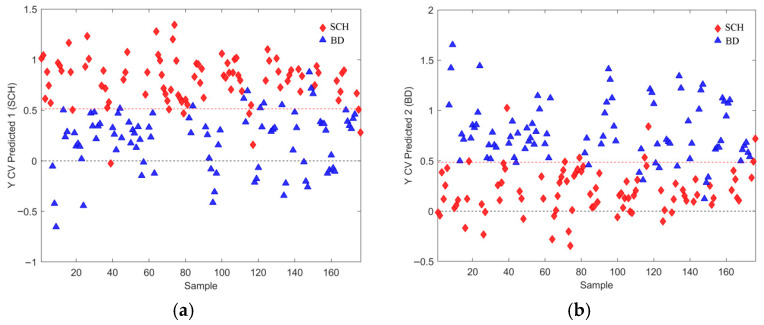
Cross-validated prediction results for the complete dataset and selected features from subset 2, given in Table 1: (**a**) class SCH (red filled diamonds), where the discrimination threshold was determined at 0.5150; (**b**) class BD (blue filled triangles), where the discrimination threshold was determined at 0.4850.

**Figure 11:**
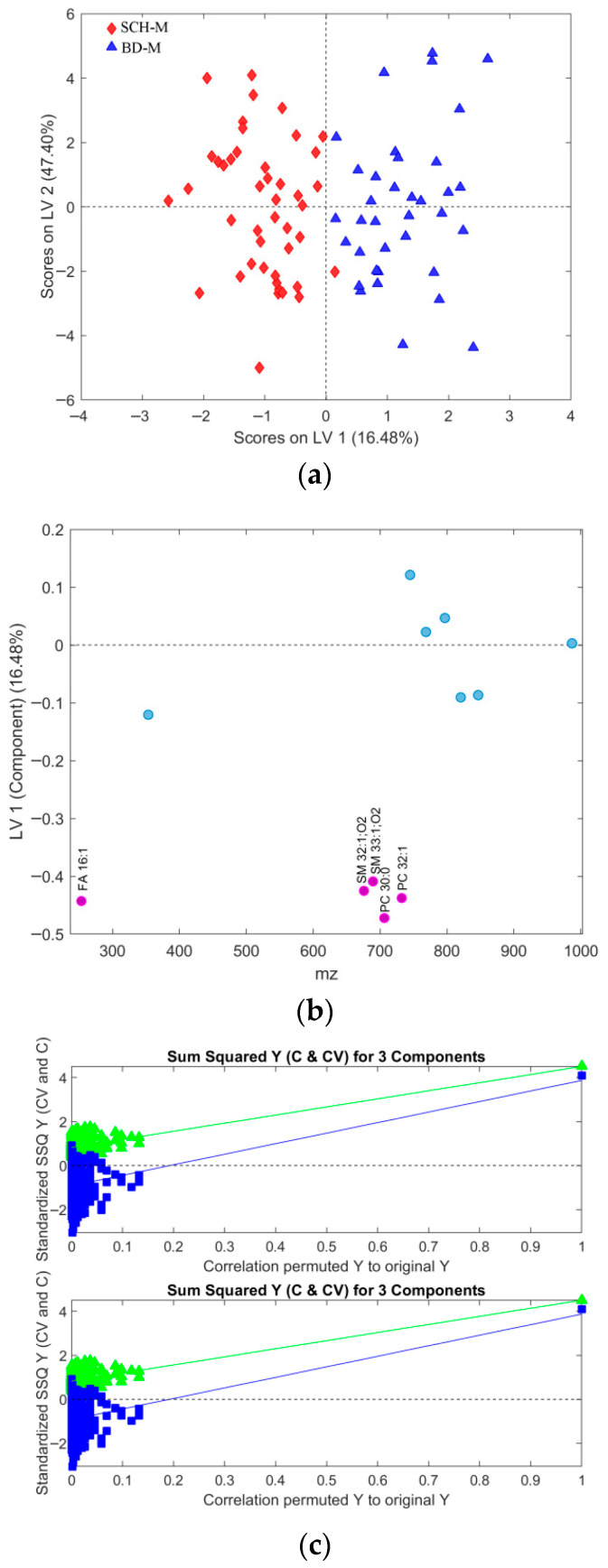
(**a**) Scores plot of the first two LV components of the OPLS-DA model using mean centering and unit variance scaling for only male subjects in the initial dataset. Variance captured by first two LV components, predictive LV1 and orthogonal LV2, is given in the figure axis labels. (**b**) Loading LV1 component of the PLS-DA model for the set of features assigned as subset 4 (given in Table 2), with the relevant subset of features including only male subjects from the initial list of samples with identified outliers excluded, where features with VIP scores greater than 1 are indicated as pink circles assigned with corresponding names. (**c**) Permutation test for the OPLS-DA model composed of three LV components and the set of selected features assigned as subset 4, performed with 500 iterations. Fractional y-block information captured by calibration (green) and cross-validation (blue) versus y-correlation is shown.

**Figure 12:**
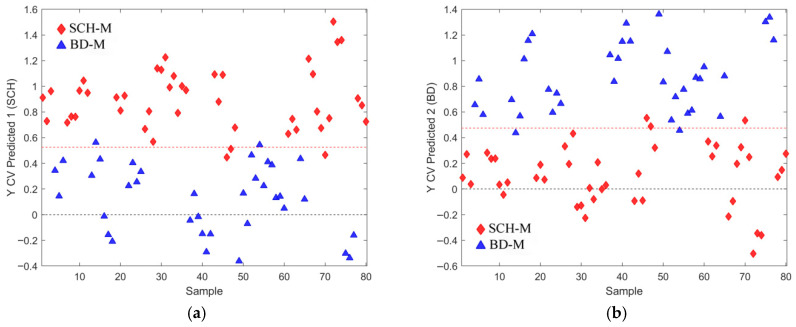
Cross-validated prediction results for male subjects only and selected features from subset 4, given in Table 2: (**a**) class SCH-M (red filled diamonds), where the discrimination threshold was determined at 0.5255 (red dashed line); (**b**) class BD-M (blue filled triangles), where the discrimination threshold was determined at 0.4745 (red dashed line).

**Figure 13:**
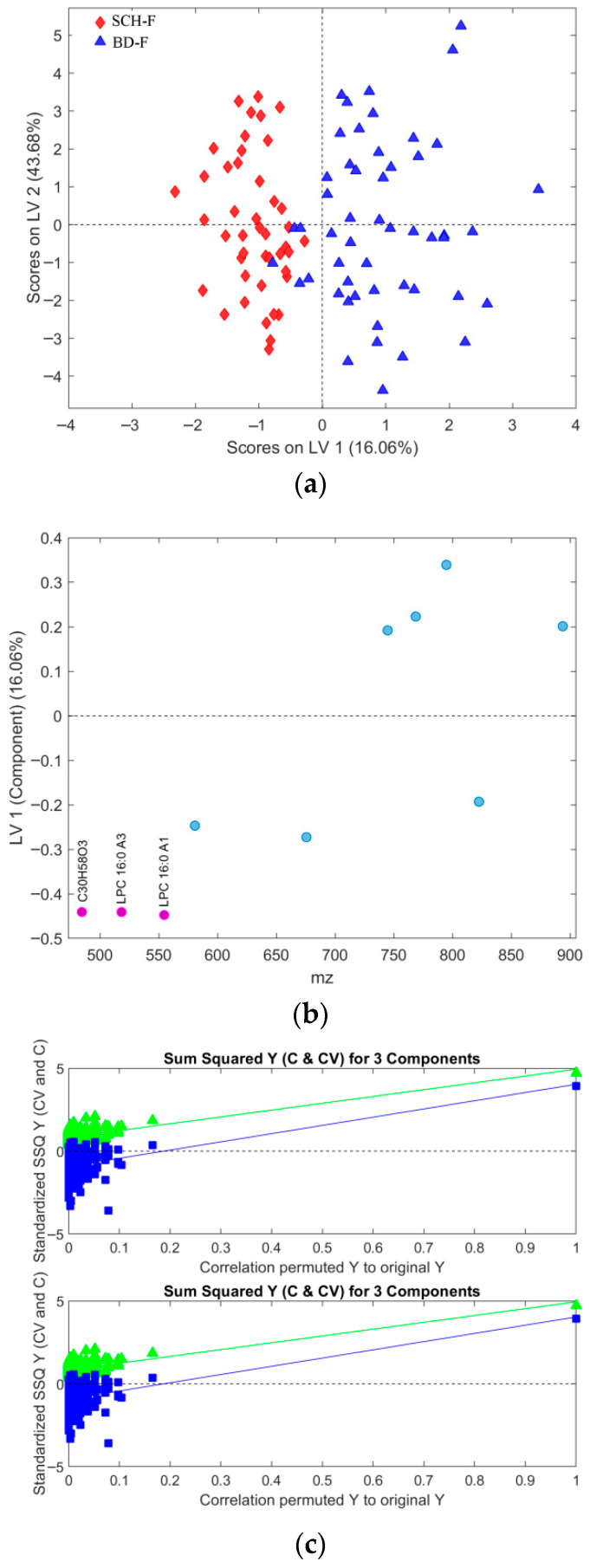
(**a**) Scores plot of first the two LV components of the OPLS-DA model using mean centering and unit variance scaling for only female subjects in the initial dataset. Variance captured by the first two LV components, predictive LV1 and orthogonal LV2, is given in the figure axis labels. (**b**) Loading LV1 component of the PLS-DA model for the set of features assigned as subset 6 (given in Table 3), with the relevant subset of features including only female subjects from the initial list of samples with identified outliers excluded, where features with VIP scores greater than 1 are indicated as pink circles assigned with corresponding names. (**c**) Permutation test for the OPLS-DA model composed of three LV components and the set of selected features assigned as subset 2, performed with 500 iterations. Fractional y-block information captured by calibration (green) and cross-validation (blue) versus y-correlation is shown.

**Figure 14:**
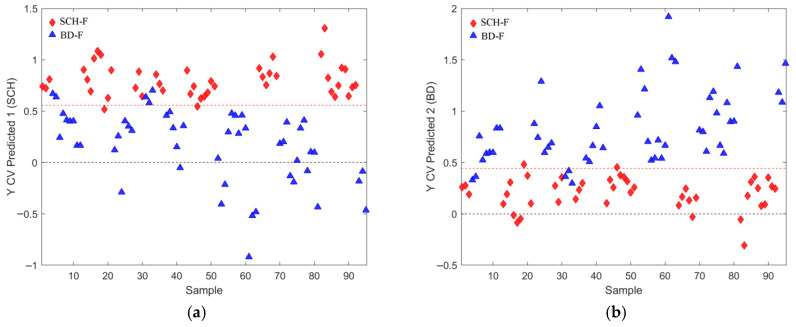
Cross-validated prediction results for female subjects only, and selected features from subset 6 given in Table 3: (**a**) class SCH-F (red filled diamonds), where the discrimination threshold was determined at 0.5581 (red dashed line); (**b**) class BD-F (blue filled triangles), where the discrimination threshold was determined at 0.4419 (red dashed line).

The best OPLS-DA models rely on the relevant feature subsets (subsets 2, 4, and 6 in Table 1, Table 2 and Table 3) are visualized in Figure 9, Figure 10, Figure 11, Figure 12, Figure 13 and Figure 14 with scores plots for the first two LV components (predictive and orthogonal) and loading plots, highlighting the most relevant features (VIP scores > 1). Permutation tests (using 500 iterations) were performed to address potential overfitting [73,74].

Model performance metrics in Figures S9–S11 (Supplementary Materials) align with Table 5, showing significant improvements for models with selected features compared to those obtained with all features. Comparison of Figure 2a vs. Figure 9a reveals pronounced sample shifts (“SCH” and “BD”) from the central coordinate line separating two classes along the LV1 predictive component, supporting this observation.

In the dataset with all observations, the best OPLS-DA model’s loading plot (Figure 9b) identified the following features based on the “greater than one rule”: “LPC 16:0 A1”, “SM 33:1;O2”, “SM 32:1;O2”, “C30H58O3”, “PC 30:0”, and “*m*/*z* 293.1779”. These features are ranked in increasing order, as shown in Table 1.

In the male-only dataset (Figure 11b), the OPLS-DA model using subset 4 (Table 2) identified five key features (VIP > 1): “SM 32:1;O2”, “PC 32:1”, “SM 33:1;O2”, “PC 30:0”, and “FA 16:1”.

For the female-only dataset (Figure 13b), three features were identified: “LPC 16:0 A1”, “LPC 16:0 A3”, and “C30H58O3”. The feature “Cer 36:2;O3,” despite ranking higher than “C30H58O3,” did not meet the VIP > 1 criterion for subset 6 (Table 3). All identified features appear in the top three positions of subset 5 for the same table.

A comparison of feature lists from OPLS-DA models across male, female, and all-observation datasets revealed limited overlap. Common features include “SM 32:1;O2,” “SM 33:1;O2,” and “PC 30:0” for males, and “LPC 16:0 A1” and “C30H58O3” for females, appearing in the full dataset as well. Each significant feature (VIP > 1) was specific to its gender group.

However, Table 5 shows that models with all observations perform worse than gender-specific models. This may result from interactions between features favoring one gender, causing confounding effects in combined datasets. These findings suggest that gender-based partitioning improves OPLS-DA model performance, like datasets containing SCH and C (control) groups [19].

On the other hand, a key limitation of this study is its relatively small dataset, which, as a consequence, prevents the partitioning of the independent subset needed for external validation with a feasible number of observations. Moreover, the dependency of classification tasks of the so-called wrapper method, which evaluates subsets of features based on the selected classifier performance, unavoidably leads to biased results, depending on the modeling algorithm on which they were evaluated [75]. To improve reliability in such circumstances, an additional modeling algorithm should be applied to confirm the selected features.

**Figure 15:**
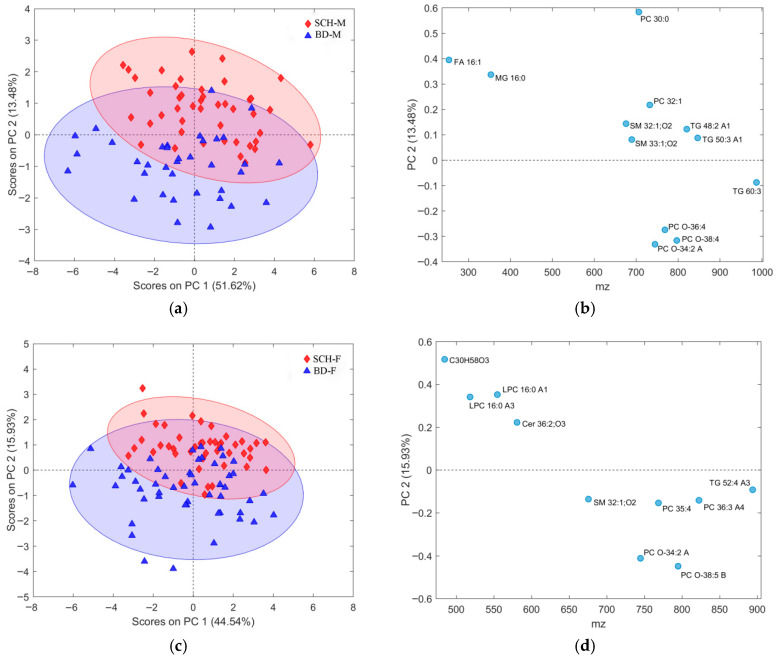
PCA model for the dataset partition with: (**a**) male subjects and the selected subset of features, using four PCA components with the explained 84.28% overall variability; (**b**) corresponding loading plot of the PC2 component of the dataset partition with male subjects and the selected subset of features; (**c**) female subjects and the selected subset of features, using four PCA components with the explained 78.51% overall variability; and (**d**) corresponding loading plot of the PC2 component of the dataset partition with female subjects and the selected subset of features. Each group of patients was assigned a different color (BD as blue full triangles and SCH as red full diamonds), where each feature in (**b**,**d**) was separately assigned a corresponding code name.

Besides the extensive application of CV used in all stages during the procedure of selection and subsequent validation of each subset of features in this work, Worley and Powers, based on their findings [58], also suggested using PCA models as a comparative practical indicator of OPLS-DA model reliability. Thus, if the procedure for feature selection followed in this study is acceptable, then we should expect to observe, from the assembled PCA models, more distinguishable group separation belonging to the recognized class of patients, in both partitions of datasets. This will also confirm the statistical significance of the class separation observed in the obtained OPLS-DA models. Initial PCA models for gender-specific lists of observations (Figure 1) showed no clear group separation, but models using the best subset of selected features (Figure 15) revealed distinct separation along PC2. The explained variance increased from 8.57% to 13.48% in males and from 12.42% to 15.93% in females, confirming the relevance of the selected features. Increased variance between patient groups also confirms the relevance of each selected feature subset, as independently validated by both OPLS-DA and PCA models. Furthermore, the PC2 loading plots (Figure 15b,d) closely matched those from OPLS-DA models (Figure 11b and Figure 13b), reinforcing consistency across methods.

## 4. Discussion

Although PCA of gender-differentiated subgroups (SCH-M vs. BD-M and SCH-F vs. BD-F) did not reveal distinct separation patterns (Figure 1), the most relevant feature subset pairs—1 and 2 for all samples, 3 and 4 for male subgroups, and 5 and 6 for female subgroups—were robustly validated using optimal metrics from a 1000-fold cross-validated OPLS-DA model. Among these, subsets 2, 4, and 6 represent the smallest feature sets with the best metric performance for the overall, male, and female datasets, respectively (Table 1, Table 2 and Table 3; Figure 9, Figure 10, Figure 11, Figure 12, Figure 13 and Figure 14). The OPLS-DA model applied to all samples (SCH vs. BD) using the minimal feature subset 2 identified six potential biomarkers: LPC 16:0, SM 33:1, SM 32:1, compound C30H58O3, PC 30:0, and an unidentified compound with *m*/*z* 293.1779, with VIP scores higher than 1 (Table 1, Figure 9b). Gender-differentiated models revealed five biomarkers in the male subgroups (SCH-M vs. BD-M): sphingomyelins SM 32:1 and SM 33:1, phosphatidylcholines PC 32:1 and PC 30:0, and FA 16:1 (Table 2, Figure 11b), and two biomarkers in the female subgroups (SCH-F vs. BD-F): two forms of lysophosphatidylcholine LPC 16:0 (A1 and A3, representing different adducts) and compound C30H58O3 (Table 3, Figure 13b), suggesting sex-specific lipidomic signatures that are also supported by the literature data [19,76,77,78,79]. Tabassum et al. (2023) highlighted that genetic factors, such as sex chromosomes and physiological differences like menopause and sex hormones, may contribute to gender-specific lipidomic profiles [79]. These findings suggest that lipid metabolism regulation differs between men and women, and blood lipidomics (serum and plasma) should be analyzed separately by gender, treating it as a confounding factor [76,77,78,79]. Actually, our results showed that in male patients (SCH-M vs. BD-M), three of five potential biomarkers (SM 32:1, SM 33:1, and PC 30:0) are common with those found in the overall SCH vs. BD comparison, while in female patients (SCH-F vs. BD-F), both of the identified biomarkers (two forms of LPC 16:0 and C30H58O3) are also present in the overall SCH vs. BD comparison (Table 1, Table 2 and Table 3, Figure 9, Figure 11 and Figure 13). Moreover, potential biomarkers appear to be distinct between female and male patients with no overlap.

Obviously, comparative analysis of lipid profiles between SCH and BD groups including all samples, as well as between their respective male and female subgroups, confirmed alterations in lipid metabolism, including three main lipid classes: sphingophosholipids (SPs), glycerophospholipids (GPs), and free fatty acids (FAs), which were also observed in our previous studies of the lipidomic profiles of SCH and BD patients compared to controls [19,20]. SPs and GPs are fundamental components of brain membranes. Sphingomyelins (SMs) are particularly important for transmembrane signaling, while GPs contribute significantly to the structural integrity and fluidity of neuronal membranes [80,81]. Our findings clearly indicate alterations in both GP (including PC 32:1, PC 30:0, and LPC 16:0) and SP (including SM 32:1 and SM 33:1) metabolic pathways, pointing to a disruption in lipid homeostasis. This imbalance likely affects membrane architecture and intracellular signaling mechanisms, which may underline the pathophysiology of SCH and BD. The potential biomarkers identified include lipid species from the classes of PC, SM, and LPC. According to the literature data, PC and SM are generally elevated in women, while LPC is higher in men, at least partly due to biological sex differences in the activities of phospholipases and the synthesis of SMs [76,77,78,79]. Since lipid species belonging to classes of PC, SM, and LPC could be influenced both by gender and psychiatric disorders, it could be crucial to perform gender-specific analysis when searching for disorder-specific biomarkers of the psychiatric disorders.

Only a few studies [18,82,83,84] have simultaneously analyzed the lipidomic profiles of plasma and serum in both BD and SCH patients, although in half of them [64,65], comparisons between patient cohorts and the control group were not presented as primary outcomes. Costa et al. (2023) found that most differential lipids between BD and SCH were SPs (54.17%), followed by GLs and GPs (16.67% each), with sterol lipids and FA being the least common [82]. Their plasma lipidomics analysis suggests that focusing on these lipid classes could help distinguish between BD and SCH, which agrees with our results. On the other hand, however, Tkachev et al. (2023) found that changes in plasma lipidomic profiles associated with SCH and BD are largely similar [18]. One of the main findings was that alterations in ceramides (Cer) levels were characteristic of both BD and SCH patients. However, Cer (d32:1) and Cer (d38:1) levels were dysregulated in BD patients but not in SCH patients compared to healthy controls, and changes in serum Cer (d43:3) were specific to SCH [18].

Yu et al. (2024) used plasma lipidomics data from 7145 Finnish individuals to study the association of five psychiatric disorders, including SCH and BD, with plasma lipid profiles [84]. They identified 179 lipid species across 13 classes. In BD patients, several phosphatidylcholine (PC) lipid species were strongly correlated with disease, with some showing protective effects and others linked to higher risk. For SCH patients, 22 lipid species, mainly from the GP class, were associated with the diagnosis.

Tao et al. (2022) analyzed the lipidomic profiles of 112 SCH patients, 132 BD patients, 105 MDD patients, and 198 healthy controls [83]. They aimed to identify psychosis subtypes rather than differences from healthy controls. Using the UHPLC-MS method, 1164 lipid molecules were identified, with 10 key lipids distinguishing psychotic patients from controls. Seven lipids were upregulated (mostly derivatives of PCs and FA) and three were downregulated (derivatives of vitamin D3, an unsaturated FA, and gamma-butyric acid (GBA)). Patients were classified into two subtypes (Cluster 1 and Cluster 2) based on these lipids, and 66% of SCH and 48% of BD patients were included in Cluster 2, with lower global assessment scores and significant white brain matter alterations compared to Cluster 1 patients. This suggests that lipid biomarkers can identify transdiagnostic subtypes across psychiatric diseases.

Our analysis of lipid profiles between SCH and BD groups, especially considering the common potential biomarkers in gender-differentiated groups (SCH-M vs. BD-M and SCH-F vs. BD-F) with those found in the overall SCH vs. BD comparison (Table 1, Table 2 and Table 3, Figure 9, Figure 11 and Figure 13), suggests that diagnosis-specific alterations in lipid metabolism in SCH and BD may be closely linked to the modulation or upregulation of phospholipase A2 (PLA2) enzyme activity. PLA2 plays a catalytic role in hydrolyzing GPs, producing corresponding unsaturated fatty acids and lysophospholipids. Elevated PLA2 activity has previously been reported in SCH and is strongly associated with changes in neuronal function, which may contribute to affective and cognitive symptoms [80]. The lipidomic biomarkers identified in our study, particularly those consistently altered in both gender-differentiated groups (SCH-M vs. BD-M and SCH-F vs. BD-F), highlight the significance of enzymes regulating these metabolites.

In the overall comparison (SCH vs. BD), all lipid putative biomarkers (LPC 16:0, SM 33:1, SM 32:1, compound C30H58O3, and PC 30:0) showed higher abundance in SCH compared to BD, except for *m*/*z* 293.1779, which was decreased in SCH compared to BD patients (Table 1, Figure 9b). Similarly, our results of male patients (SCH-M vs. BD-M) confirmed that all potential biomarkers, SM 32:1, SM 33:1, PC 32:1, PC 30:0, and FA 16:1, were more abundant in SCH-M than BD-M (Table 2, Figure 11b). In addition, both of the identified biomarkers (two forms of LPC 16:0 and C30H58O3) in female patients (SCH-F vs. BD-F) had elevated levels in SCH-F compared to BD-F (Table 3, Figure 13b). To the best of our knowledge, the literature-available data for the comparison of the serum lipidomics profiles of SCH and BD patients are limited and inconsistent. Costa et al. (2023) found that most differential lipids belonging to SP, GL, and GP were downregulated in SCH compared to BD and healthy controls [80], which is the opposite of our results. Tkachev et al. (2023) found no differences in choline and ethanolamine-containing GPs and TGs in BD patients, suggesting these changes might be more pronounced in SCH patients [18], which agrees with our results. Yu et al. (2024) confirmed the importance of PC lipid alterations in both BD and SCH, suggesting that their positive association might be more pronounced in BD [84], which is the opposite of our results.

Finally, the limitations of this study are very important to emphasize. The study limitations include a moderate sample size (n = 61—SCH 30 and 31 BD), which may affect statistical power. Additionally, the results were obtained using a semi-quantitative method without full validation against reference standards. Although sex differences were considered, other confounding factors, such as dietary habits, lifestyle, comorbidities, duration of illness, and the use of first- and second-generation antipsychotics, may influence GP and SP content, and should be addressed in future studies incorporating rigorous control or stratification for these factors [85]. Therefore, in future research, larger multicenter studies are essential to validate these potential biomarkers across diverse populations.

## 5. Conclusions

This study demonstrates the utility of advanced lipidomic profiling in addressing one of the most persistent challenges in psychiatry, the differential diagnosis of schizophrenia (SCH) and bipolar disorder (BD). Using LC-HRMS combined with robust chemometric modeling, we identified distinct lipid signatures that reliably discriminate between these two disorders. The validated feature subsets, pairs 1 and 2, captured the most relevant metabolites differentiating SCH and BD patients, identifying five key biomarkers: LPC 16:0, SM 33:1, SM 32:1, compound C30H58O3, and PC 30:0. Gender-specific models further revealed five biomarkers in males (SM 32:1, SM 33:1, PC 32:1, PC 30:0, and FA 16:1) and two in females (LPC 16:0 and C30H58O3), highlighting potential sex-related metabolic differences. The potential biomarkers, primarily glycerophospholipids and sphingophospholipids, not only highlight metabolic differences between SCH and BD, but also reveal gender-specific patterns, underscoring the importance of personalized approaches in psychiatric research.

The elevated levels of these lipid classes in SCH patients suggest disruptions in membrane lipid homeostasis and signaling pathways, yet the precise mechanistic links between these lipids and disease pathophysiology remain unclear. Functional studies, including pathway analyses and experimental validation, are necessary to elucidate these relationships. The lack of validation in independent cohorts or functional assays represents a critical gap for clinical application, highlighting the need for future replication studies and targeted metabolomic assays to confirm biomarker reliability and assess their predictive value for treatment response and disease progression.

## Figures and Tables

**Table 2:** For the male subset of samples, the list of identified relevant features for subset 3, consisting of the 20 most relevant features from the 37 overall features in this subset; and for subset 4, consisting of the 12 most relevant features from the 29 total features in this subset.

Subset 3		Subset 4	
ID Number	Feature Names	ID Number	Feature Names
106	SM 32:1;O2	106	SM 32:1;O2
39	PC 32:1	39	PC 32:1
107	SM 33:1;O2	107	SM 33:1;O2
37	PC 30:0	37	PC 30:0
16	FA 16:1	16	FA 16:1
5	Cer 34:1;O2 B	93	PC O-34:2 A
93	PC O-34:2 A	35	MG 16:0
46	PC 34:0	99	PC O-38:4
35	MG 16:0	132	TG 48:2 A1
99	PC O-38:4	143	TG 50:3 A1
51	PC 34:2 A2	97	PC O-36:4
129	TG 48:1 A1	183	TG 60:3
59	PC 36:2 A1		
149	TG 51:2 A1		
118	SM 40:2;O2		
132	TG 48:2 A1		
143	TG 50:3 A1		
166	TG 54:5 A1		
97	PC O-36:4		
183	TG 60:3		

**Table 3:** For the female subset of samples, the list of identified relevant features for subset 5, consisting of the 9 most relevant features from the 15 total features in this subset; and for subset 6, consisting of the 10 most relevant features from the 21 total features in this subset.

Subset 5		Subset 6	
ID Number	Feature Names	ID Number	Feature Names
24	LPC 16:0 A1	24	LPC 16:0 A1
26	LPC 16:0 A3	26	LPC 16:0 A3
1	C30H58O3	8	Cer 36:2;O3
101	PC O-38:5 B	1	C30H58O3
57	PC 35:4	101	PC O-38:5 B
158	TG 52:4 A2	93	PC O-34:2 A
178	TG 56:8 A1	57	PC 35:4
66	PC 36:3 A4	159	TG 52:4 A3
155	TG 52:3 A2	106	SM 32:1;O2
		66	PC 36:3 A4

**Table 4:** Skewness for classification metrics presented for all distributions obtained from 1000 CV OPLS-DA models for each dataset (including all observations assigned as ds, and solely with males assigned as ds_male or females assigned as ds_female participants), obtained by using all and selected (subset 2, 4, or 6) lists of features (appended to the base name for the corresponding group of samples following snake_case convention) for their assembly.

Skewness	ds_All	ds_ Selected	ds_Male_ All	ds_Male_ Selected	ds_Female_ All	ds_Female_ Selected
accuracy	−0.3355	−0.5339	−0.6120	−1.1246	−0.4323	−1.1702
AUROC	−0.6971	−0.7312	−1.2077	−2.5998	−0.5925	−1.2864
sensitivity	−0.4327	−0.4533	−0.7758	−1.0454	−0.4366	−1.1509
specificity	−0.1993	−0.8569	−0.5685	−1.1961	−0.4632	−1.7518

**Table 5:** The best values for accuracy, AUROC, sensitivity, and specificity obtained among 1000 generated CV OPLS-DA models are given for each processed dataset (including the complete dataset, assigned as ds, and with only males assigned as ds_male or females assigned as ds_female), with all and corresponding relevant feature subsets appended to the base name for the corresponding group of samples (following the snake_case convention) given in Table 1, Table 2 and Table 3.

Feature Subsets	Accuracy	AUROC	Sensitivity	Specificity	Number of Features
ds_all	0.8523	0.9401	0.8523	0.8523	183
ds_subset1	0.9091	0.9654	0.9318	0.8864	16
ds_subset2	0.8977	0.9551	0.9091	0.8864	14
ds_male_all	0.8625	0.9590	0.8636	0.8611	183
ds_male_subset3	0.9125	0.9867	0.9091	0.9167	20
ds_male_subset4	0.9375	0.9956	0.9318	0.9444	12
ds_female_all	0.8211	0.9314	0.8182	0.8235	183
ds_female_subset5	0.8947	0.9652	0.9091	0.8824	9
ds_female_subset6	0.9263	0.9857	0.9545	0.9020	10

## Data Availability

The lipidomics data presented in this study are unavailable due to privacy or ethical restrictions.

## Supplementary Materials

The following supporting information can be downloaded at: https://www.mdpi.com/article/10.3390/metabo16010069/s1, Figure S1. (a) Boxplot of log10-transformed data for the schizophrenia (SCH) and bipolar disorder (BD) classes of patients. (b) Boxplot of log10-transformed and mean centered data for the same classes. Figure S2. PCA model obtained for four consecutive batches. Figure S3. ROBPCA outlier map of the LC-MS dataset composed using: (a) four PC components for the whole dataset including all observations of both classes; (b) three PC components for the subset including only male participants of SCH and BD classes; and (c) four PC components for the subset including only female participants of SCH and BD classes. Figure S4. Results for: (a) skewness and (b) kurtosis of the log10 transformed raw data before and after the removal of identified outliers. The x-axis shows *m*/*z* (mass-to-charge ratio), while the y-axis displays related statistics for any variable in the dataset. Figure S5: Histogram of frequency counts obtained from: (a) the minimum of RMSECV; (b) the minimum of class error; and (c) the maximum of AUROC in all OPLS-DA models applied on 1000 subsets of randomly selected triplicates for each sample of each of the 5 folds. The maximum number of LV components in all models was 10. The applied CV method for the presented results was “venetian blinds”. Figure S6. Permutation test performed with 500 iterations for the best OPLS-DA model depicted in Figure 4, selected among 1000 CV models with a complete set of observations with (a) three LV components and (b) five LV components. The sum of squares of responses for the calibrated models are presented in green, while the CV models are presented in blue. Figure S7. Performance metrics displayed in relation to the increasing sequence of sets of ranked features for partition of the initial dataset containing male subjects only: (a) accuracy; (b) AUROC; (c) class error; and (d) RMSECV. Additionally, markers colored as green squares (subset 3) and red circles (subset 4) represent the smallest sets of features identified through the selection procedure. The first two features were omitted, since the number of LV components was always 3 for each of the assembled OPLS-DA models during validation. Both subsets with corresponding feature coding lists are presented in Table 2. Figure S8. Performance metrics displayed in relation to the increasing sequence of sets of ranked features for partition of the initial dataset containing female subjects only: (a) accuracy; (b) AUROC; (c) class error; and (d) RMSECV. Additionally, markers colored as green squares (subset 5) and red circles (subset 6) represent the smallest sets of features identified through the selection procedure. The first two features were omitted, since the number of LV components was always 3 for each of the assembled OPLS-DA models during validation. Both subsets with corresponding feature coding lists are presented in Table 3. Figure S9. Comparison of the performance metric distribution of 1000 assembled CV OPLS-DA models of the complete dataset of all included features (given in blue), and with the subset of relevant selected features assigned as subset 2 in Table 1 (given in red), for: (a) accuracy; (b) AUROC; (c) sensitivity; and (d) specificity. At the same time, for each presented distribution, the median for the given metric distribution was assigned a dashed line in a corresponding color. Histograms in the distributions of each metric are given with equal bin width. Figure S10. Comparison of the performance metric distribution of 1000 assembled CV OPLS-DA models of the subset of data including male subjects only, with all included features (given in blue) and with the subset of relevant selected features assigned as subset 4 in Table 2 (given in red), for: (a) accuracy; (b) AUROC; (c) sensitivity; and (d) specificity. At the same time, for each presented distribution, the median for the given metric distribution was assigned a dashed line in a corresponding color. Histograms in the distributions of metrics are given with equal bin width.; Figure S11. Comparison of the performance metric distribution of 1000 assembled CV OPLS-DA models of the subset of data including female subjects only, with all included features (given in blue), and with the subset of relevant selected features assigned as subset 6 in Table 3 (given in red), for: (a) accuracy; (b) AUROC; (c) sensitivity; and (d) specificity. At the same time, for each presented distribution, the median for the given metric distribution was assigned a dashed line in a corresponding color. Histograms in the distributions of metrics are given with equal bin width. Table S1: Identification of relevant features for subsets 1–6 (Table 1, Table 2 and Table 3) relating to the lipids found differently in all, male, and female schizophrenia and bipolar disorder patients.
