# Supplementary material for: Discrimination of Bipolar Disorder and Schizophrenia Patients Based on LC-HRMS Lipidomics
Source: Metabolites. 2026 Jan 12;16(1):69. doi: 10.3390/metabo16010069 (PMC12843932; doi:10.3390/metabo16010069)
Supplement: Supplementary file 1 [file metabolites-16-00069-s001.zip › metabolites-4012736-supplementary.pdf]

Supplementary Materials

Article

Discrimination of Bipolar Disorder and Schizophrenia Patients Based on LC-HRMS Lipidomics

Milan R. Janković [1], Nataša Avramović [2,*], Zoran Miladinović [3], Milka B. Jadranin [4], Marija Takić [5], Gordana Krstić [1], Aleksandra Gavrilović [6], Čedo Miljević [7], Maja Pantović [8], Zorana Andrić [9], Savvas Radević [9], Danica Savić [4], Stefan Lekić [4], Vele Tešević [1] and Boris Mandić [1,*]

[1] Faculty of Chemistry, University of Belgrade, Studentski Trg 12–16, 11000 Belgrade, Serbia
[2] Faculty of Medicine, Institute of Medical Chemistry, University of Belgrade, Višegradska 26, 11000 Belgrade, Serbia
[3] Institute of General and Physical Chemistry, University of Belgrade, Studentski Trg 12–16, 11158 Belgrade, Serbia
[4] Department of Chemistry, Institute of Chemistry, Technology and Metallurgy, University of Belgrade, Njegoševa 12, 11000 Belgrade, Serbia
[5] Group for Nutrition and Metabolism, Center of Research Excellence for Nutrition and Metabolism, Institute for Medical Research, National Institute of Republic of Serbia, University of Belgrade, Tadeuša Košćuška 1, 11000 Belgrade, Serbia
[6] Special Hospital for Psychiatric Diseases "Kovin", Cara Lazara 253, 26220 Kovin, Serbia
[7] Faculty of Medicine, Institute of Mental Health, University of Belgrade, Milana Kašanina 3, 11000 Belgrade, Serbia
[8] Clinic of Psychiatry, University Clinical Center of Serbia, Pasterova 2, 11000 Belgrade, Serbia
[9] Blood Transfusion Institute of Serbia, Svetog Save 39, 11000 Belgrade, Serbia
* Correspondence: natasa.avramovic@med.bg.ac.rs (N.A.); borism@chem.bg.ac.rs (B.M.)

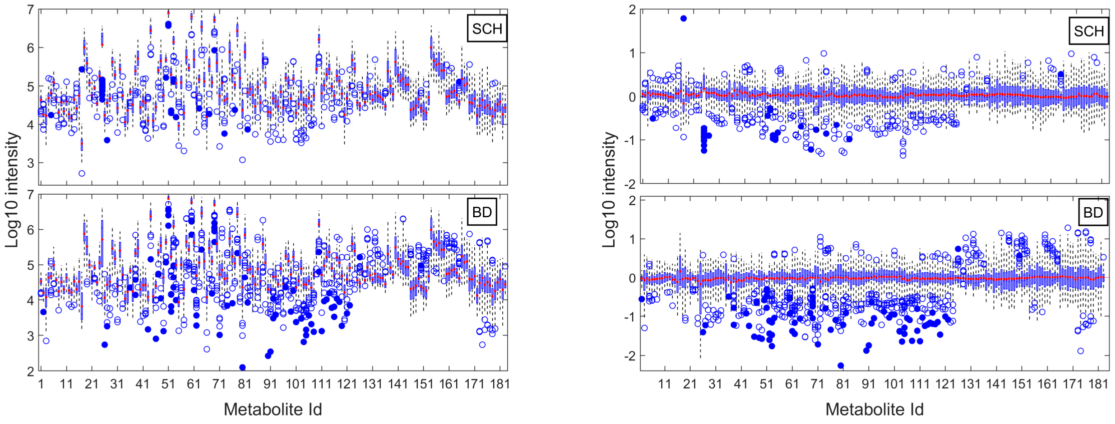

(**a**) (**b**)

FigureS1. (**a**) Boxplot of log10-transformed data for classes Schizophrenia (SCH) and Bipolar disorder (BD) group of patients; (**b**) Boxplot of log10-transformed and mean centered data for the same classes.

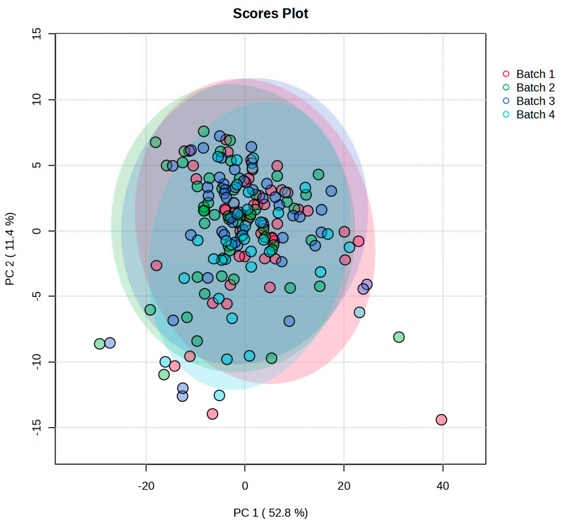

Figure S2. PCA model obtained for four consecutive batches.

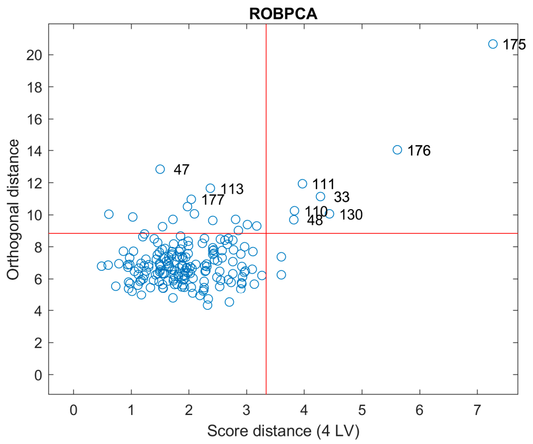

(**a**)

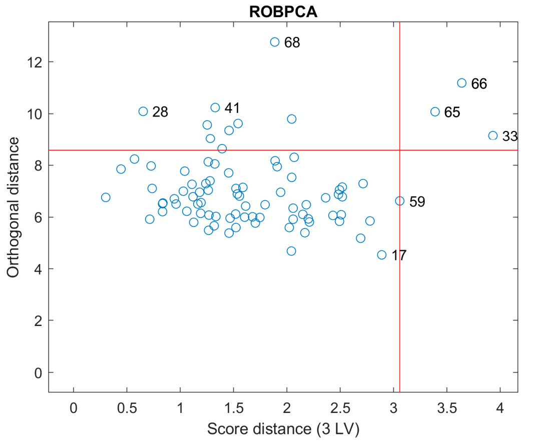

(**b**)

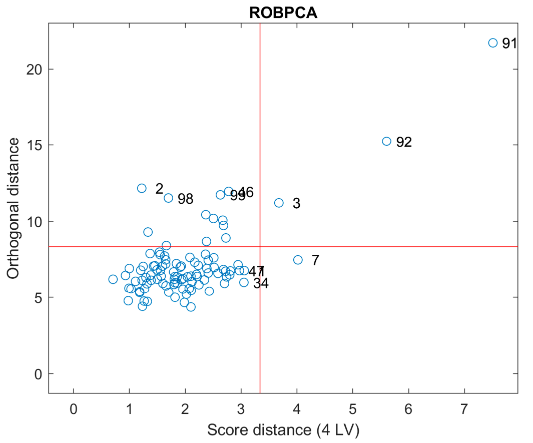

(**c**)

Figure S3. ROBPCA outlier map of LC-MS data set composed using: (**a**) four PC components for whole data set, including all observation of both classes; (**b**) three PC components for sub-set including only male participants of SCH and BD classes; (**c**) four PC components for sub-set including only female participants of SCH and BD classes.

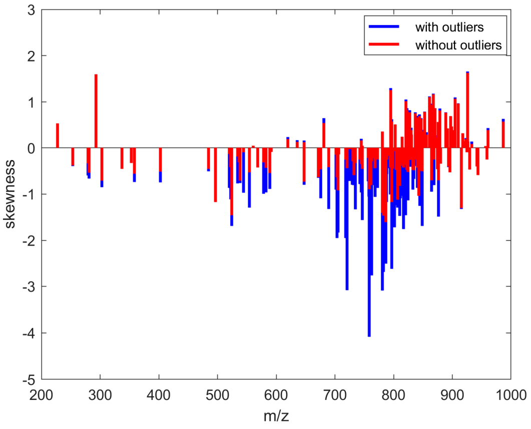

(**a**)

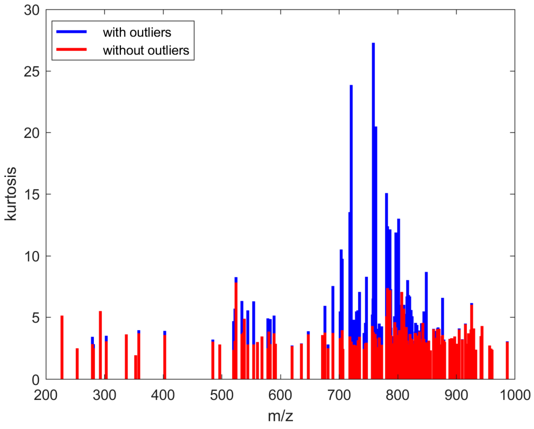

(**b**)

Figure S4. Results for: (**a**) skewness and (**b**) kurtosis of log10 transformed raw data before and after removal of identified outliers. On the x-axis was given *m/z* (mass-to-charge ratio), while on the y-axis was displayed related statistics for any variable in data set.

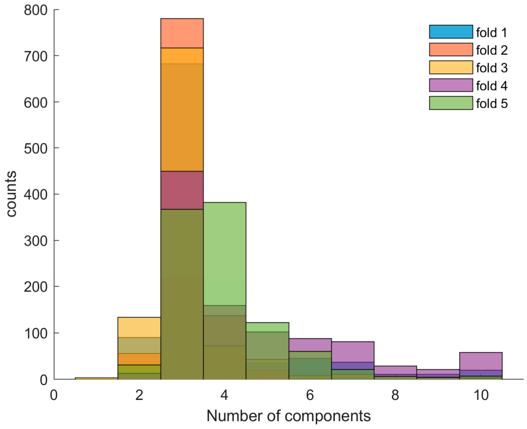

(**a**)

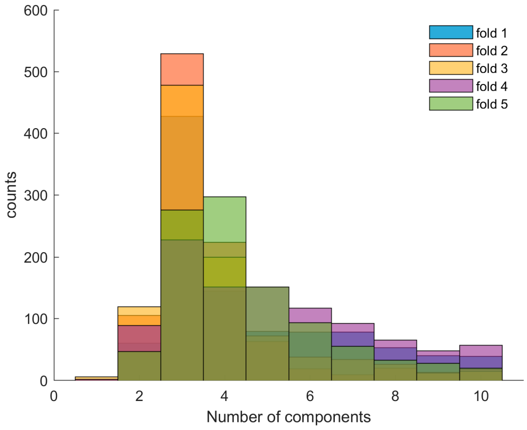

(**b**)

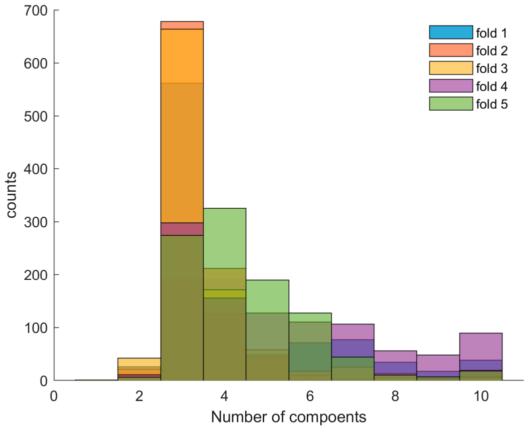

(**c**)

Figure S5. Histogram of frequency counts obtained from: (**a**) minimum of RMSECV; (**b**) minimum of class error; **(c)** maximum of AUROC in all OPLS-DA models applied on 1000 subsets of randomly selected triplicates for each sample of each of the 5 folds. Maximum number of LV components in all models was 10. Applied CV method for presented results was “venetian blind”.

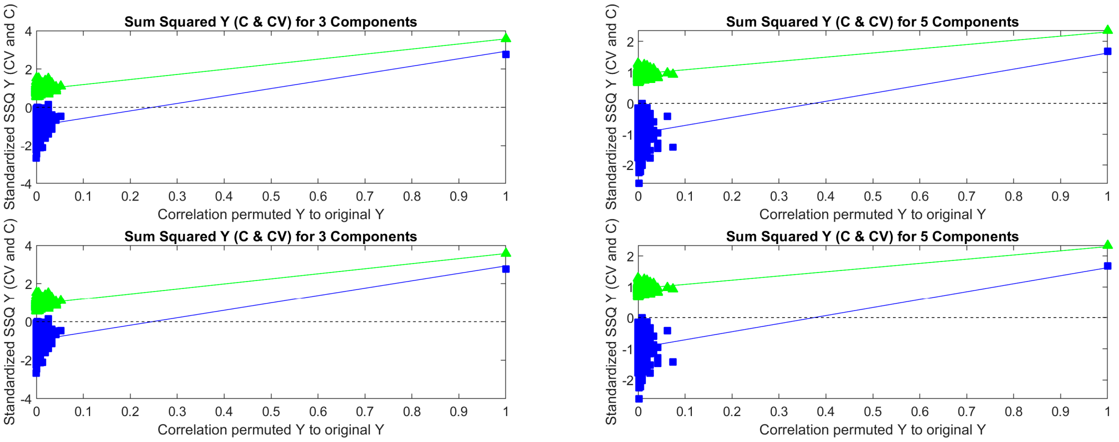

(**a**) (**b**)

Figure S6. Permutation test performed with 500 iterations for best OPLS-DA model depicted in Figure 4 selected among 1000 CV models with complete set of observation with (**a**) three LV components and (**b**) five LV components. Sum of squares of responses for calibrated models was presented in green, while CV models were presented in blue.

Features selection methods and algorithm

To ensure robust variable ranking, k-fold partition resampling (without replacement) was applied to create different training set versions [19]. This process is structured into following three main stages:

Feature Selection Stages

1. Datasets (complete and gender-partitioned) were re-partitioned into 5-fold subgroups of samples, keeping triplicates intact. Five independent calibration sets were created by omitting one-fold each time, repeated *iter1* times.
2. For each calibration subset, random sub-sampling was performed by selecting one replicate per individual. This step was repeated *iter2* times, ensuring each patient contributed only one replicate to the final subset.
3. After *iter1* and *iter2* iterations, the resulting structure included *iter1* × *iter2* × *fold* subsets, where fold = 5.
4. Regular OPLS-DA models were built for each subset using a predefined number of LV components. VIP scores from each model were recorded in a matrix of size (*iter1* × *iter2* × *fold, p)*, where *p* = number of features.
5. Variables were ranked in descending order of VIP scores for each model, with their indices recorded in a ranking matrix of the same size as the VIP scores matrix.
6. A matrix containing sorted VIP values (aligned with the ranking matrix) was also generated.
7. Two matrices of size (*iter1* × *iter2* × *fold, p)* were produced: one for feature rankings and the other for associated VIP scores.

Global Feature Ranking

In the next stage, all *iter1* × *iter2* × *fold* ranked lists from the previous step were aggregated to create a global ranked list, reducing the matrix size from (*iter1* × *iter2* × *fold, p)* to a vector of dimension (*1, p*).

1. *Borda Count Method*: This voting-theory algorithm assigns a score to each variable based on the sum of features with higher positions across the *iter1* × *iter2* × *fold* lists. The global ranked list was then generated by sorting features according to their Borda count, which corresponds to their average rank across all lists [37,67].
2. *Frequency-Based Ranking*: In parallel, features satisfying VIP > 1 in the sorted VIP scores matrix (from step 6 in the previous stage) were identified. Ranking positions for these features were retrieved from the corresponding ranking matrix (step 5). Features were ranked stepwise based on their selection frequency [68], with higher frequencies resulting in higher scores.

Both methods were alternated, and the best-performing approach was used to generate the feature validation plot in the next step.

Validation of global/final/resulting ranking lists stage

Resulting ranking list of features, obtained from the previous aggregation procedure, could be presented as an order set of features *S*:

$$S = \langle s_1, s_2, s_3, \cdots, s_p \rangle$$

where the *p* represents total number of features in data set.

From this list of ordered features, *p* subsets (nested sequences) of ordered features lists could be accomplished:

Let ⟨*Sk*⟩ be a nested sequence of *p* subsets of *S* such that:

$$\langle S_k \rangle, \{\forall k \in N : 1 \leqslant k \leqslant p\}, S_k \subseteq S_{k+1}$$

For each feature subset ⟨*Sk*⟩ (*k* being the current number of features), an increasing sequence of ranked feature sets was created. For each of subset, 1000 independent OPLS-DA models were built

using all samples from the dataset, with each model independently cross-validated using a 5-fold CV partition.

Classification performance metrics (or diagnostic statistics) were calculated for each OPLS-DA model and averaged across CV values for each metric. These averages were plotted as single points on the corresponding performance plots. The dependence of averaged classification metrics (derived from CV confusion matrices) and RMSECV on increasing sets of ranked features was visualized.

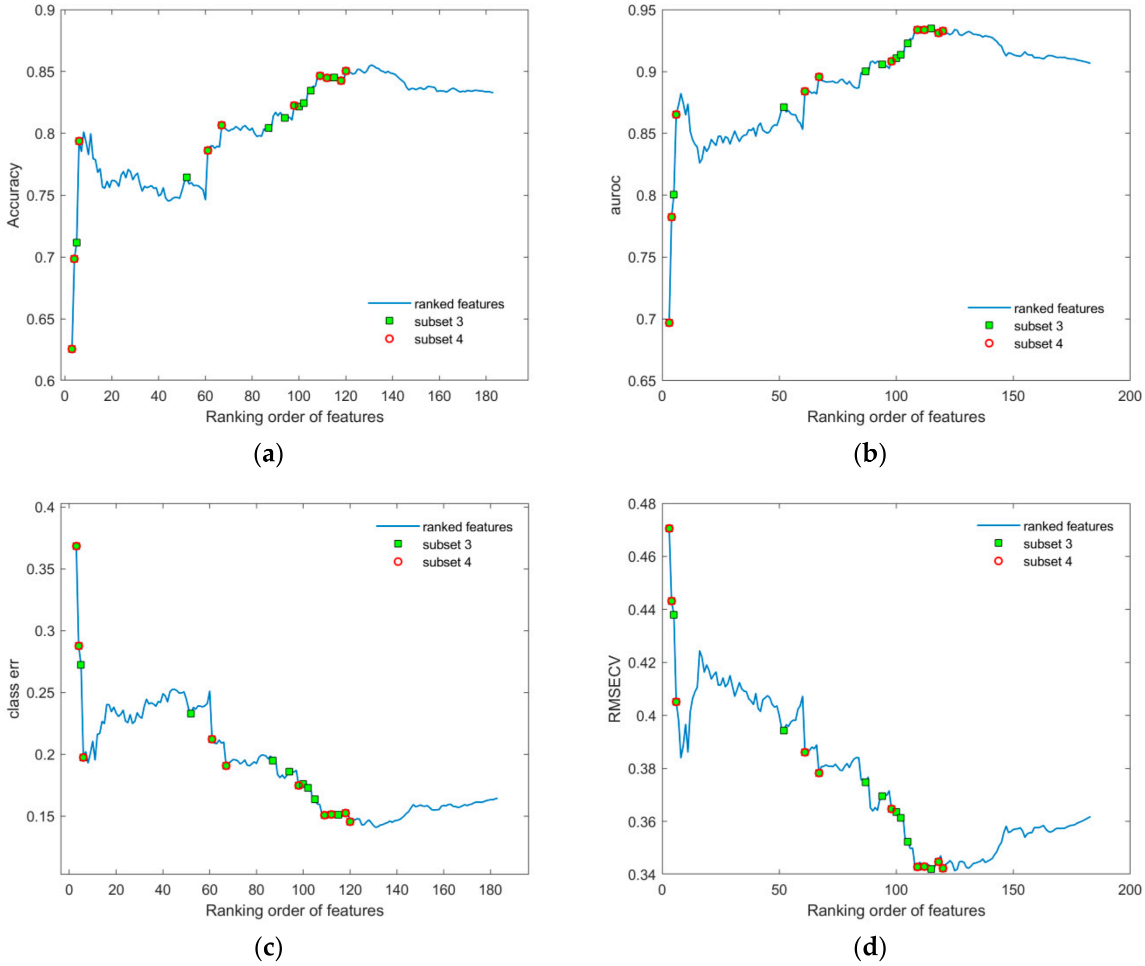

Figure S7. Performances metrics displayed in relation to the increasing sequence of sets of ranked features for partition of initial data set containing male subjects only: (**a**) accuracy; (**b**) AUROC; (**c**) class error; and (**d**) RMSECV. Additionally, markers colored in green squares (subset 3) and red circles (subset 4) represent smallest sets of features identified through selection procedure. First two features were omitted since the number of LV components was always three for each of assembled OPLS-DA models during validation. Both subsets with corresponding features coding lists were presented in Table 2.

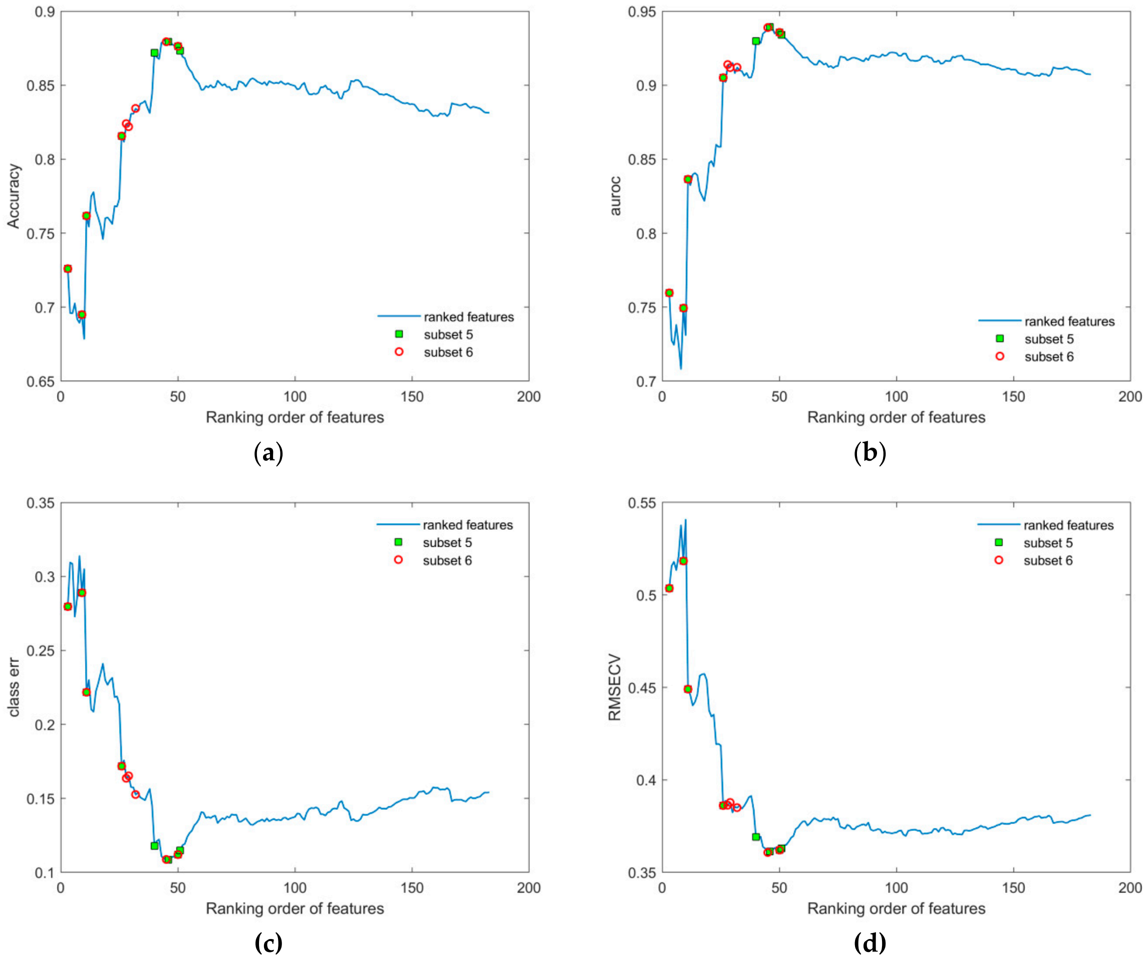

Figure S8. Performances metrics displayed in relation to the increasing sequence of sets of ranked features for partition of initial data set containing female subjects only: (**a**) accuracy; (**b**) AUROC; (**c**) class error; and (**d**) RMSECV. Additionally, markers colored in green squares (subset 5) and red circles (subset 6) represent smallest sets of features identified through selection procedure. First two features were omitted since the number of LV components was always three for each of assembled OPLS-DA models during validation. Both subsets with corresponding features coding lists were presented in Table 3.

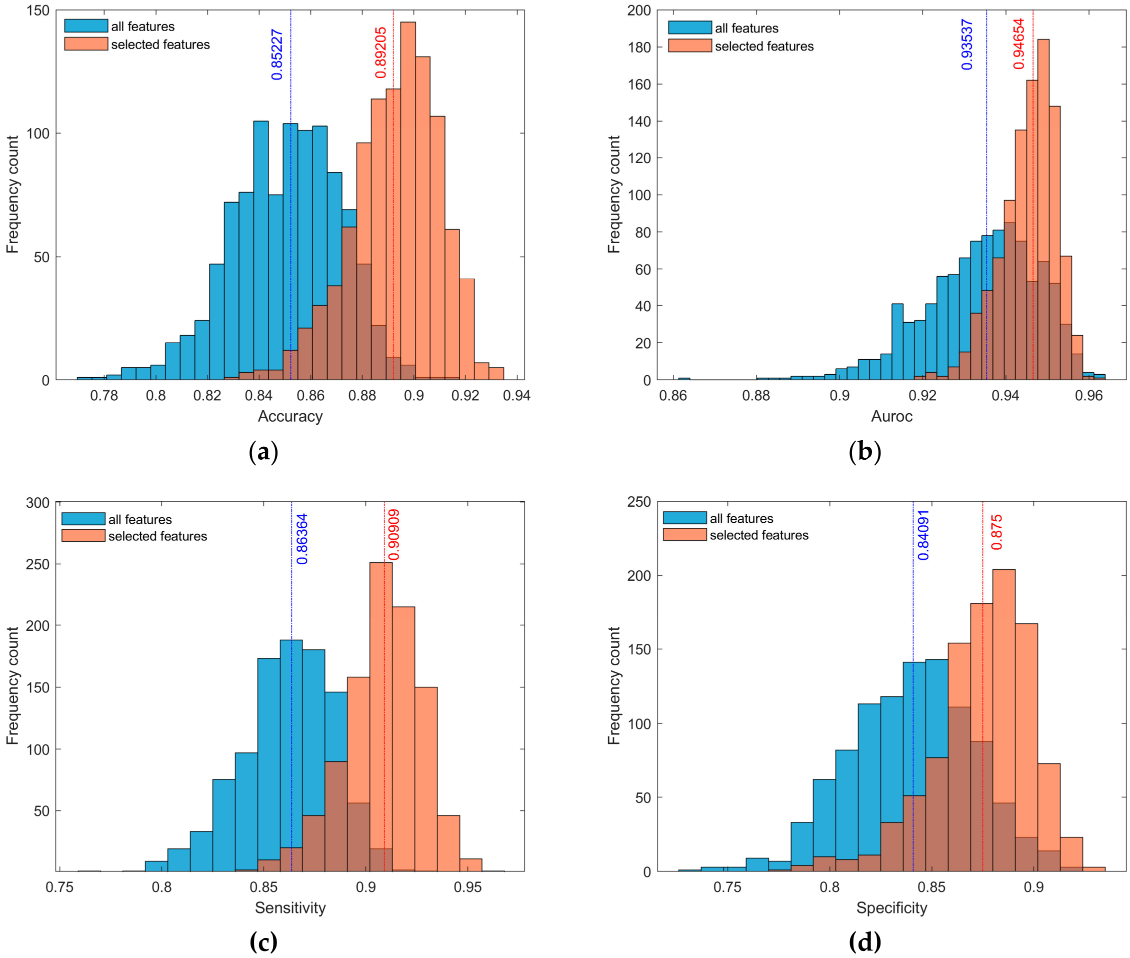

Figure S9. Comparison of performances metrics distribution of 1000 assembled CV OPLS-DA models of complete data set with all included features (given in blue) and with subset of relevant selected features assigned as subset 2 in Table 1 (given in red) for: (**a**) accuracy; (**b**) AUROC; (**c**) sensitivity; and (**d**) specificity. At the same time, for each of presented distribution, the median for given metric distribution was assigned with dashed line in corresponding color. Histograms in distributions of each metric were given with equal bin width.

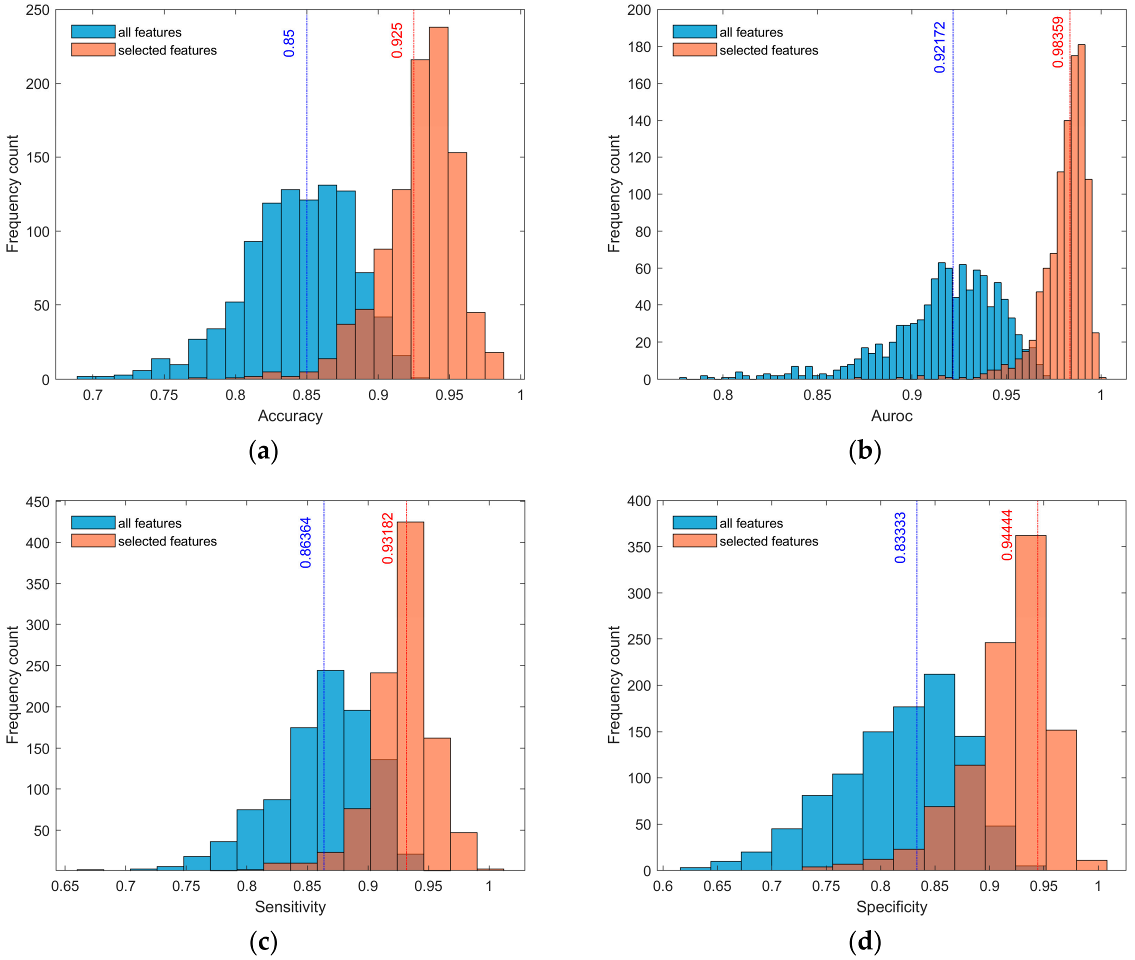

Figure S10. Comparison of performances metrics distribution of 1000 assembled CV OPLS-DA models of subset of data included male subjects only, with all included features (given in blue) and with subset of relevant selected features assigned as subset 4 in Table 2 (given in red) for: (**a**) accuracy; (**b**) AUROC; (**c**) sensitivity; and (**d**) specificity. At the same time, for each of presented distribution, the median for given metric distribution was assigned with dashed line in corresponding color. Histograms in distributions of metric were given with equal bin width.

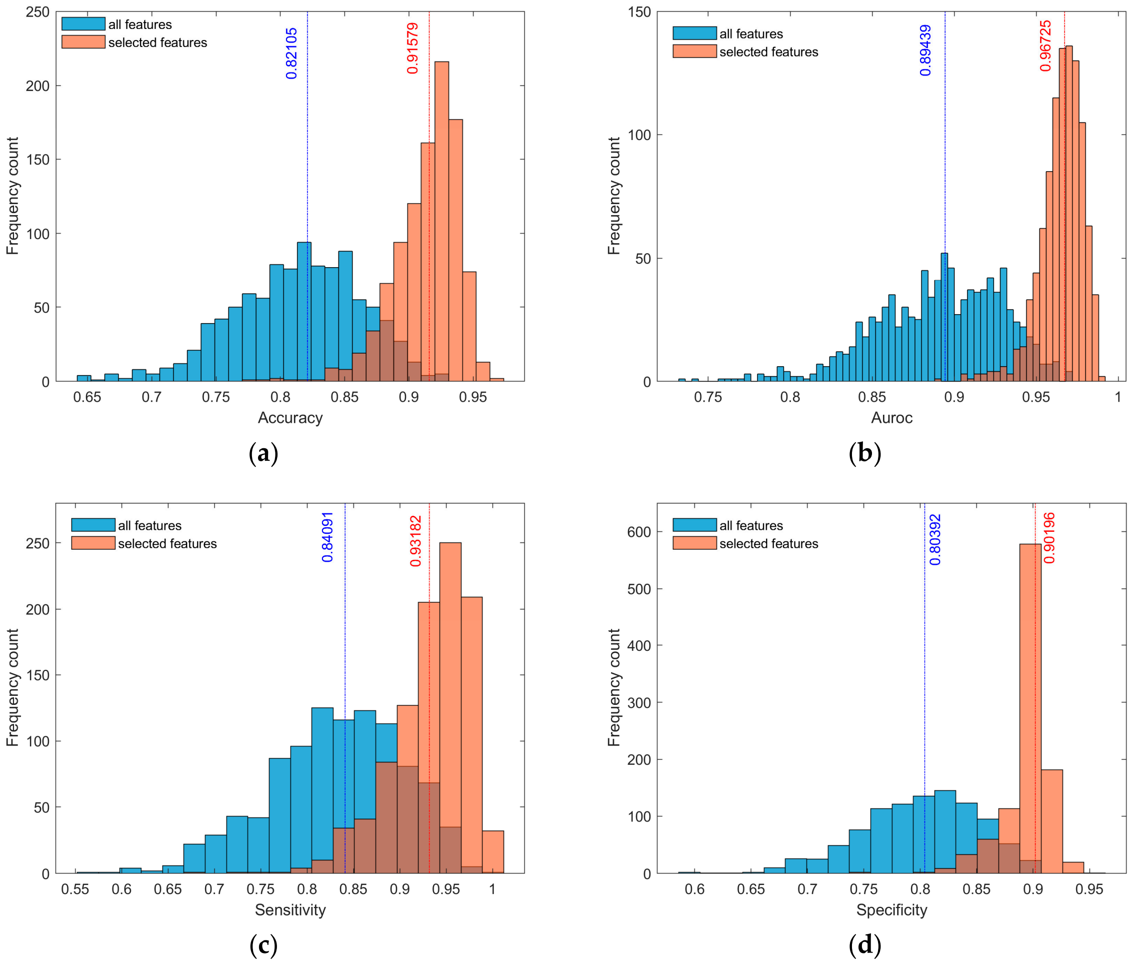

Figure S11. Comparison of performances metrics distribution of 1000 assembled CV OPLS-DA models of subset of data included female subjects only, with all included features (given in blue) and with subset of relevant selected features assigned as subset 6 in Table 3 (given in red) for: (**a**) accuracy; (**b**) AUROC; (**c**) sensitivity; and (**d**) specificity. At the same time, for each of presented distribution, the median for given metric distribution was assigned with dashed line in corresponding color. Histograms in distributions of metric were given with equal bin width.

Table S1. Identification of relevant features for subsets 1–6 (Tables 1–3) relating to the lipids found differently in all, male and female schizophrenia and bipolar disorder patients.

No.	Retention Time (min)	Feature Assignment	Measured *m/z*	Ion Mode Adduct	Proposed Formula	Lipid Assignment	Lipid Class
1	0.48	*m/z* 293.1779	293.1779	N.D.	N.D.	N.D.	N.D.
2	1.49	LPC 16:0 A1<br>LPC 16:0 A3	554.3496<br>518.3202	$[M+OAc]^-$<br>$[M+Na]^+$	$C_{24}H_{50}NO_7P$	LPC 16:0	GP
3	2.04	FA 16:1	253.2187	$[M–H]^-$	$C_{16}H_{30}O_2$	FA 16:1	FA
4	3.35	MG 16:0	353.2644	$[M+Na]^+$	$C_{19}H_{38}O_4$	MG 16:0	GL
5	4.52	C30H58O3	484.4709	$[M+NH_4]^+$	$C_{30}H_{58}O_3$	N.D.	N.D.
6	4.66	Cer 36:2;O3	580.5288	$[M+H]^+$	$C_{36}H_{69}NO_4$	Cer 36:2;O3	SP
7	5.09	SM 32:1;O2	675.5427	$[M+H]^+$	$C_{37}H_{75}N_2O_6P$	SM 32:1;O2	SP
8	5.39	Cer 34:1;O2 B	538.5181	$[M+H]^+$	$C_{34}H_{67}NO_3$	Cer 34:1;O2	SP
9	5.43	SM 33:1;O2	689.5584	$[M+H]^+$	$C_{38}H_{77}N_2O_6P$	SM 33:1;O2	SP
10	5.63	PC 38:6 A	806.5691	$[M+H]^+$	$C_{46}H_{80}NO_8P$	PC 38:6	GP
11	5.92	PC 30:0	706.5374	$[M+H]^+$	$C_{38}H_{76}NO_8P$	PC 30:0	GP
12	5.97	PC 32:1	732.5537	$[M+H]^+$	$C_{40}H_{78}NO_8P$	PC 32:1	GP
13	6.12	PC 34:2 A2	758.5787	$[M+H]^+$	$C_{42}H_{80}NO_8P$	PC 34:2	GP
14	6.32	PI 40:3	915.5997	$[M–H]^-$	$C_{49}H_{89}O_{13}P$	PI 40:3	GP
15	6.33	PC 36:3 A4	822.5400	$[M+K]^+$	$C_{44}H_{82}NO_8P$	PC 36:3	GP
16	6.37	PC O-36:5	766.5744	$[M+H]^+$	$C_{44}H_{80}NO_7P$	PC O-36:5	GP
17	6.53	PC O-36:4	768.5900	$[M+H]^+$	$C_{44}H_{82}NO_7P$	PC O-36:4	GP
18	6.64	PC O-34:2 A	744.5895	$[M+H]^+$	$C_{42}H_{82}NO_7P$	PC O-34:2	GP
19	6.83	PC 36:2 A1	786.6031	$[M+H]^+$	$C_{44}H_{84}NO_8P$	PC 36:2	GP
20	7.00	PC 35:4	768.5532	$[M+H]^+$	$C_{43}H_{78}NO_8P$	PC 35:4	GP
21	7.14	PC O-38:5 B	794.6051	$[M+H]^+$	$C_{46}H_{84}NO_7P$	PC O-38:5	GP
22	7.31	PC O-38:4	796.6209	$[M+H]^+$	$C_{46}H_{86}NO_7P$	PC O-38:4	GP
23	7.36	PC 34:0	762.5997	$[M+H]^+$	$C_{42}H_{84}NO_8P$	PC 34:0	GP
24	7.46	SM 40:2;O2	785.6529	$[M+H]^+$	$C_{45}H_{89}N_2O_6P$	SM 40:2;O2	SP
25	11.15	TG 56:8 A1	920.7693	$[M+NH4]^+$	$C_{59}H_{98}O_6$	TG 56:8	GL
26	11.25	TG 48:2 A1	820.7384	$[M+NH4]^+$	$C_{51}H_{94}O_6$	TG 48:2	GL
		TG 50:3 A1	846.7544	$[M+NH4]^+$			

27	11.27	TG 50:3 A2	851.7097	$[M+Na]^+$	$C_{53}H_{96}O_6$	TG 50:3	GL
28	11.31	TG 52:4 A2<br>TG 52:4 A3	877.7257<br>893.6996	$[M+Na]^+$<br>$[M+K]^+$	$C_{55}H_{98}O_6$	TG 52:4	GL
29	11.33	TG 54:5 A1	898.7857	$[M+NH4]^+$	$C_{57}H_{100}O_6$	TG 54:5	GL
30	11.45	TG 48:1 A1	822.7542	$[M+NH4]^+$	$C_{51}H_{96}O_6$	TG 48:1	GL
31	11.47	TG 50:2 A1	848.7704	$[M+NH4]^+$	$C_{53}H_{98}O_6$	TG 50:2	GL
32	11.49	TG 52:3 A2	879.7414	$[M+Na]^+$	$C_{55}H_{100}O_6$	TG 52:3	GL
33	11.55	TG 49:1	836.7694	$[M+NH4]^+$	$C_{52}H_{98}O_6$	TG 49:1	GL
34	11.57	TG 51:2 A1	862.7852	$[M+NH4]^+$	$C_{54}H_{100}O_6$	TG 51:2	GL
35	11.67	TG 60:3	986.9101	$[M+NH4]^+$	$C_{63}H_{116}O_6$	TG 60:3	GL

* *m*/*z* mass-to-charge ratio; FA: fatty acids; LPC: 1-acyl-sn-glycero-3-phosphocholines; GP: glycerophospholipids; MG: monoradylglycerols; GL: glycerolipids; Cer: N-acylsphinganines (dihydroceramides); SP: sphingolipids; SM: ceramide phosphocholines (sphingomyelins); PC: diacylglycerolphosphocholines; PA: glycerophosphoinositols; PC O-: 1-alkyl,2-acylglycerophosphocholines; TG: triacylglycerols; N.D. not determined.
